# Somatic genome editing with the RCAS-TVA-CRISPR-Cas9 system for precision tumor modeling

**DOI:** 10.1038/s41467-018-03731-w

**Published:** 2018-04-13

**Authors:** Barbara Oldrini, Álvaro Curiel-García, Carolina Marques, Veronica Matia, Özge Uluçkan, Osvaldo Graña-Castro, Raul Torres-Ruiz, Sandra Rodriguez-Perales, Jason T. Huse, Massimo Squatrito

**Affiliations:** 1 Seve Ballesteros Foundation Brain Tumor Group, Cancer Cell Biology Program, Spanish National Cancer Research Center, CNIO, 28029 Madrid, Spain; 2 Genes, Development, and Disease Group, Cancer Cell Biology Program, Spanish National Cancer Research Centre, CNIO, 28029 Madrid, Spain; 3 Bioinformatics Unit, Structural Biology and Biocomputing Programme, CNIO, 28029 Madrid, Spain; 4 Molecular Cytogenetics Group, Human Cancer Genetics Program, Spanish National Cancer Research Center, CNIO, 28029 Madrid, Spain; 5 Departments of Pathology and Translational Molecular Pathology, University of Texas MD Anderson Cancer Center, Houston, TX 77030 USA

## Abstract

To accurately recapitulate the heterogeneity of human diseases, animal models require to recreate multiple complex genetic alterations. Here, we combine the RCAS-TVA system with the CRISPR-Cas9 genome editing tools for precise modeling of human tumors. We show that somatic deletion in neural stem cells of a variety of known tumor suppressor genes (*Trp53*, *Cdkn2a*, and *Pten*) leads to high-grade glioma formation. Moreover, by simultaneous delivery of pairs of guide RNAs we generate different gene fusions with oncogenic potential, either by chromosomal deletion (*Bcan*-*Ntrk1*) or by chromosomal translocation (*Myb-Qk*). Lastly, using homology-directed-repair, we also produce tumors carrying the homologous mutation to human BRAF V600E, frequently identified in a variety of tumors, including different types of gliomas. In summary, we have developed an extremely versatile mouse model for in vivo somatic genome editing, that will elicit the generation of more accurate cancer models particularly appropriate for pre-clinical testing.

## Abstract (web summary)

Accurate recapitulation of human disease in animal models requires generation of complex and heterogeneous genetic variation. Here the authors combine RCAS-TVA with CRISPR-Cas9 to generate mouse models of cancer.

## Introduction

A decade of studies has underlined the complexity of the genetic events that characterize the genomic landscapes of common forms of human cancer^1^. While a few genes are mutated at high frequencies (>20%), the greatest number of cancer genes in most patients appear at intermediate frequencies (2–20%) or lower^2^. A current high priority in cancer research is to functionally validate candidate genetic alterations that are relevant for cancer progression and treatment response. In order to do so, it is essential to develop flexible models that can speed up the identification of cancer driver genes among the large number of passenger alterations^3^.

The growing level of sophistication of the genome engineering technologies has made it possible to manipulate nearly any candidate gene in vivo. The CRISPR–Cas system has revolutionized research by facilitating accurate manipulation of the genome. CRISPR-Cas has been applied in cancer modeling to the inactivation of tumor suppressor genes^4–7^, the generation of somatic point mutations^4,5,8^ and complex genomic rearrangements, such as gene fusion events^9–11^.

The RCAS-TVA-based approach uses replication-competent avian leukosis virus splice-acceptor (RCAS) vectors to target individual cells engineered to express the cell surface receptor TVA. The RCAS-TVA system has been successfully used in transgenic mice to deliver genes or shRNAs into a plethora of cell types: neural stem cells (NSCs), astrocytes, hepatocytes and pancreatic acinar cells, among many others^12^.

Here we describe a series of mouse models that combine the genome editing capability of the CRISPR-Cas9 system with the somatic gene delivery of the RCAS-TVA approach. To prove the efficacy of such a powerful system, we produced a number of glioma models with tailored genetic alterations.

We took advantage of the previously developed *Rosa26-LSL-Cas9* (*LSL-Cas9*) knockin mouse strain^4^ and combined it with the *Nestin-tv-a* (*Ntv-a*) and the *GFAP-tv-a* (*Gtv-a*) transgenic mice that express the TVA receptor under the control of the rat *nestin* and human *GFAP* promoter, respectively^13,14^. Through in vivo delivery of RCAS plasmids that carry guide RNAs (gRNAs) for a series of tumor suppressor genes (*Trp53*, *Cdkn2a*, and *Pten*), we show that we can efficiently generate high-grade tumors in mice that express both Cas9 and TVA in the Nestin or GFAP-positive cells. Additionally, by ex vivo transduction of RCAS plasmids expressing pairs of gRNAs into NSCs, we generated either chromosomal deletion (*Bcan-Ntrk1* gene fusion) or chromosomal translocation (*Myb-Qk* gene fusion). We further show that in vivo delivery of RCAS-gRNA plasmids for the *Bcan-Ntrk1* gene fusion led to high-grade glioma tumor formation. Moreover, we generated *Braf* mutant gliomas by inducing a homology-directed repair-mediated BRAF V637E mutation, homologous to the human BRAF V600E mutation.

Lastly, by ex vivo and in vivo treatment of some of these tumor models, we demonstrate their utility for pre-clinical testing of targeted therapies.

In conclusion, by combining the RCAS-TVA and CRISPR-Cas9 models we have developed an extremely powerful mouse model for in vivo somatic genome editing, that allows targeting specific cell types with definite genetic alterations to generate precision tumor models.

## Results

### Generation of CNS Cas9-expressing mouse strains

To test the possibility of somatic genome editing by combining the RCAS-TVA and CRISPR-Cas9 models, we generated a series of mouse strains that allowed the TVA and Cas9 expression in specific cell types in the brain.

Nestin is an intermediate filament protein (IFP) that is predominantly expressed in the central nervous system stem/ progenitor cells during embryonic development^15^. In adult organism, its expression in the brain is mainly restricted to the NSC compartment of the subventricular zone (SVZ). After differentiation, nestin is downregulated and replaced by tissue-specific IFPs^16^. The glial fibrillary acidic protein (GFAP) is an IFP that in the CNS is primarily expressed by the astrocytes^17^. *Nestin-tv-a* (*Ntv-a*) and *GFAP-tv-a* (*Gtv-a*) transgenic mice, that carry the TVA receptor under the control of the rat *nestin* and human *GFAP* promoter, have been widely used for modeling brain tumorigenesis^13,14,18^.

The Rosa26-LSL-Cas9 knockin mice (*LSL-Cas9*) have a floxed-STOP cassette precluding expression of the downstream bicistronic sequences (Cas9-P2A-EGFP) and it was generated to overcome the delivery challenges of the Cas9 enzyme to specific tissues of interest^4^. We crossed these mice with the *Ntv-a* and *Gtv-a* transgenic mice to obtain the *Ntv-a; LSL-Cas9* and *Gtv-a; LSL-Cas9*. Although RCAS-Cre-expressing plasmids have been previously used in combination with different TVA-expressing mice to allow tissue-specific deletion of a variety of floxed alleles^18–20^, to ensure a robust recombination in the CNS of the floxed-STOP cassette in the *Ntv-a; LSL-Cas9* and *Gtv-a; LSL-Cas9* we further crossed these mice with either the *Nestin-Cre* (*Nes-Cre*) or *hGFAP-Cre* transgenic lines^21,22^. The resulting *Ntv-a; Nes-Cre; LSL-Cas9* and *Gtv-a; hGFAP-Cre; LSL-Cas9* mice presented no abnormalities in development and size (Supplementary Fig. 1a), were fertile and had normal litter sizes.

**Fig. 1:**
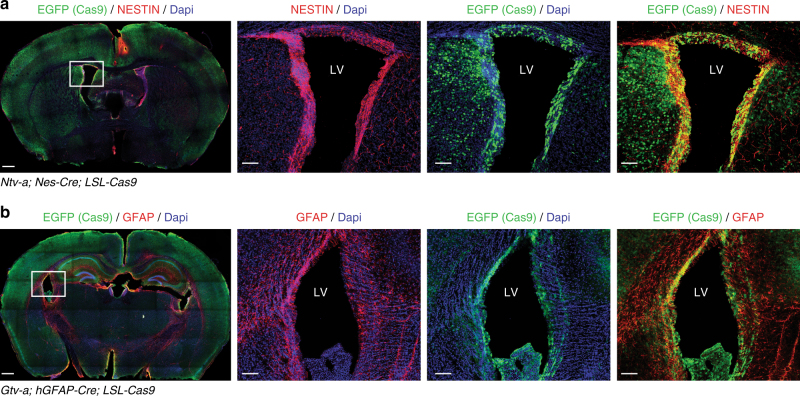
Cas9 expression in the brain of TVA/Cas9 mouse strains. **a**, **b** Immunofluorescence staining performed on brain sections of four-week-old *Ntv-a; Nes-Cre; LSL-Cas9* and *Gtv-a; hGFAP-Cre; LSL-Cas9* mice with antibody against EGFP (Cas9), NESTIN and GFAP. CAS9 is widely expressed in the whole brain and co-localize with NESTIN and GFAP in the subventricular zone. Left panels: whole brain section; right panels: higher magnification of the left panel inset. Scale bars: left panels, 500 μm; right panels, 100 μm. LV lateral ventricle

The Nestin-Cre is expressed quite early during development, beginning at E9.5, while the hGFAP-Cre appears to be expressed around E12.5-E13.5^21,22^. Both strains lead to widespread expression of the Cas9-P2A-EGFP throughout the brain of adult mice and pups (Fig. 1a, b and Supplementary Fig. 1b). Also of note it is the co-localization of NESTIN and GFAP with EGFP in the area of the sub-ventricular zone (Fig. 1a, b), one of the known site of neurogenesis of adult mice, indicating robust Cas9 expression in the NSC compartment.

### Efficient gene knockouts by RCAS-gRNA vectors

**Fig. 2:**
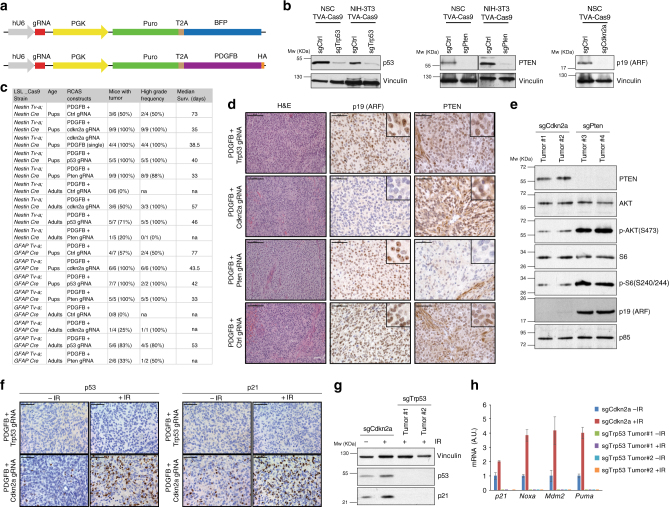
Tumor suppressor genes knockout by RCAS-gRNA plasmids induce high-grade-gliomas. **a** Schematic illustration of the RCAS-gRNA plasmids. **b** In vitro validation of the RCAS-gRNA against *Trp53*, *Pten, Cdkn2a* and a non-targeting control (Ctrl). Western blot analysis, using the indicated antibodies, on whole cell extracts from NIH-3T3 TVA-Cas9 fibroblasts and *Ntv-a; LSL-Cas9* neural stem cells (NSCs) transduced with pMSCVhygro-CRE (NSC TVA-Cas9). To induce p53 expression, the cells were collected 24 h after exposure to ionizing radiation (10 Gy). **c** Table summarizing the injections performed in the *Ntv-a; Nes-Cre; LSL-Cas9* and *Gtv-a; hGFAP-Cre; LSL-Cas9* pups and adult mice. Co-injection of RCAS-PDGFB and the RCAS-gRNA against different tumor suppressor genes accelerate tumor formation, increases the tumor penetrance and the frequency of high-grade gliomas. **d** Hematoxylin and eosin (H&E) and immunohistochemical stainings (IHCs), using the indicated antibodies, of representative RCAS-PDGFB/gRNA tumors. To note PTEN expression in the normal vasculature but not in the tumor cells of the RCAS-PDGFB + RCAS-Pten-gRNA tumor. Insets show higher magnification images. Scale bars: H&E 100 μm; IHCs 50 μm. **e** Western blot analysis, using the indicated antibodies, on whole cell extracts from tumorspheres. **f** IHCs for p53 (left panels) and p21 (right panels) on the indicated tumors 1 h after exposure to 10 Gy IR. Scale bars: 50 μm. **g** Western blot analysis, using the indicated antibodies, on whole cell extracts from tumorspheres 24 h after exposure to 10 Gy IR. **h** Quantitative real-time PCR (qPCR) analysis on mRNA extracted from tumorspheres 3 h after exposure to 10 Gy IR. Data presented as mean ± SD (*n* = 3); A.U. arbitrary unit

To explore whether the newly engineered RCAS/tv-a/Cas9 strains were suitable for in vivo genome editing, we first generated a series of RCAS plasmids that would allow the expression of gRNAs. For this purpose, we sub-cloned into the RCAS vector a cassette carrying a human U6 promoter (hU6), followed by a PGK promoter that drove the expression of a puromycin resistance gene (Puro) linked to a blue fluorescent protein (BFP) via a self-cleavable T2A peptide (hU6-gRNA-PGK-Puro-T2A-BFP) (Fig. 2a). We then cloned different previously described gRNAs^6,23,24^ targeting tumors suppressor genes (TSGs) frequently altered in high-grade gliomas: *TP53*, *CDKN2A*, and *PTEN* (mutated or deleted in 30, 62, and 41% GBM patients, respectively) (Supplementary Fig. 2a). The *CDKN2A* locus codes for two different proteins p16(INK4a) and p14(ARF) (known as p19ARF in mouse), both having tumor suppressor activity in gliomas. For our studies, we have used a gRNA targeting *Cdkn2a* exon 1β and therefore specific for p19ARF. To test the knockout efficiency of the RCAS-gRNA plasmids, we derived NSCs from *Ntv-a; LSL-Cas9* and infected them with a Cre-expressing plasmid to induce Cas9 expression. In parallel we also generated NIH-3T3 mouse fibroblasts expressing both TVA and the Cas9 genes. We then infected both cell lines with multiple rounds of infections using the various RCAS-gRNA plasmids. After either drug-selection (for the NSCs TVA-Cas9) or fluorescent-activated cell sorting (FACS) (for the BFP in the NIH-3T3 TVA-Cas9) we verified the deletion of *Trp53*, *Cdkn2a* and *Pten* by western blot analysis. Since NIH-3T3 cells bear a deletion in the *Cdkn2a* locus, we tested the *Cdkn2a* gRNA only in the NSCs. As shown in Fig. 2b, we observed efficient deletion of all those genes in both cellular systems.

Platelet-derived growth factor (PDGF) signaling is frequently aberrantly activated in GBM, primarily due to the amplification of the PDGF-receptor alpha (*PDGFRA*)^25,26^ (Supplementary Fig. 2a). RCAS-PDGFB intracranial injection into *Ntv-a* and *Gtv-a* pups has been previously shown to induce gliomas with variable penetrance (from 40 to 75%), with only a small fraction of the tumors (25%) presenting high-grade features^27–29^. Injections into *Ntv-a; Nes-Cre; LSL-Cas9* and *Gtv-a; hGFAP-Cre; LSL-Cas9* pups of either RCAS-PDGFB together with RCAS-TSG-gRNA vectors (either one of *Trp53*, *Cdkn2a* or *Pten* gRNAs), or a bicistronic RCAS-Cdkn2a-gRNA-PDGFB construct (Fig. 2a), resulted in a shortened tumor latency and increased total tumor incidence as compared to the co-injections of RCAS-PDGFB and RCAS-gRNA non-targeting control (Ctrl) (Fig. 2c and Supplementary Fig. 2b). The vast majority (80–100%) of the RCAS-TSG-gRNA-injected mice showed histological features of high-grade gliomas, including pseudopalisading necrosis, microvascular proliferation and high percentage of Ki67-positive cells (Fig. 2c, d and Supplementary Fig. 2c). Very low frequency of apoptotic cells (as measured by Cleaved caspase 3 immunostaining) was detected in both RCAS-TSG-gRNA and RCAS-Ctrl-gRNA-induced tumors (Supplementary Fig. 2c).

RCAS-PDGFB injection into adult *Ntv-a* and *Gtv-a* mice results in very low tumor penetrance (approximately 15–20%) and a quite long latency (over 100 days)^18^. Similarly, the co-injection of the RCAS-PDGFB and RCAS-Ctrl-gRNA in adult *Ntv-a; Nes-Cre; LSL-Cas9* and *Gtv-a; hGFAP-Cre; LSL-Cas9* had a considerably limited tumor potential and no tumors were observed in our 120 days’ experimental timeframe. However, similarly to what observed in pups, the injection of RCAS-PDGFB/RCAS-TSG-gRNA accelerated tumor formation, with the majority of the tumors presenting high-grade characteristics (Fig. 2c and Supplementary Fig. 2b).

Immunohistochemistry (IHC) of paraffin-embedded tumor tissue and western blot analysis of tumor-derived neurospheres (tumorspheres) showed loss of p19ARF and Pten expression in the tumors injected with the corresponding RCAS-TSG-gRNA plasmid (Fig. 2d, e). As expected, *Pten* null tumor tissues and tumorspheres presented increased AKT signaling, as evidenced by high phospho-AKT and phospho-S6 immunostaining (Supplementary Fig. 2d and Fig. 2e). Moreover, we verified that the *Cdkn2a* gRNA was targeting only p19ARF and did not affect the expression of p16(INK4a) (Supplementary Fig. 2e).

To confirm the inactivation of p53 in the RCAS-Trp53-gRNA-induced tumors, we exposed tumor-bearing mice and tumorspheres to ionizing radiation (IR): neither p53 nor p21, a well-known p53-induced target, were detectable in the *Trp53* null tumors as compared to the *Cdkn2a* null tumors (Fig. 2f, g). Furthermore, real-time quantitative PCR (qPCR) analysis on IR-treated tumorspheres showed lack of expression of a panel of p53 transcriptional targets (*p21*, *Noxa*, *Mdm2* and *Puma*) (Fig. 2h).

Sanger sequencing confirmed the presence of deletions and/or insertions at the gRNA target sites in tumorspheres from the various TSG models (Supplementary Fig. 3).

GBM expression subtypes (proneural, classical and mesenchymal) have been related to both genomic abnormalities and differences in tumor microenvironment^26,30^. *TP53* and *CDKN2A* alterations have been found to be significantly more frequent in the proneural and classical subtype, respectively^26^. Though *PTEN* deletion events were observed in all the subtypes, 100% of classical GBM showed homozygous or heterozygous deletions in the *PTEN* locus^26^. Analogously, single sample Gene Set Enrichment Analysis (ssGSEA) on RNA sequencing expression data of the RCAS-TSG tumorspheres indicated similarity to the proneural subtype for the *Trp53* null cells and to the classical subtype for both the *Cdkn2a* and *Pten* null cells (Supplementary Table 1).

In summary, these data demonstrate that RCAS-gRNA constructs can induce loss of the gene of interest in an in vivo setting and they could lead to the generations of tumors that closely resemble their human counterparts.

### Cas9 in adult mice does not activate a robust immune response

The injection of the RCAS-gRNA plasmids into *Ntv-a; Nes-Cre; LSL-Cas9* and *Gtv-a; hGFAP-Cre; LSL-Cas9* mice should lead to an early editing of the gene of interest, due to the Cas9 expression at the time of the injection. In order to have an in vivo model for time-controlled gene editing, we crossed the *Ntv-a; LSL-Cas9* mice with the *hUBC-CreERT2*^31^, for inducible Cas9 expression upon tamoxifen exposure.

There have been controversial reports of immune response to Cas9 in different experimental models^32–34^. Thus, we performed an in-depth analysis of a possible immune response in the *Ntv-a; LSL-Cas9*; *hUBC-CreERT2* upon Cas9 induction.

**Fig. 3:**
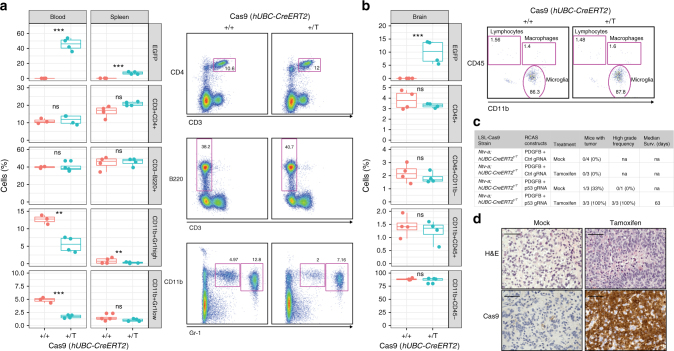
Ubiquitous Cas9 expression in TVA-Cas9 adult mice does not induce a robust immune response. **a** Left panels: Flow cytometry analysis for the specified markers in blood and spleen of *Ntv-a; LSL-Cas9*; *hUBC-CreERT2* mice of the indicated genotype. Four-week-old mice were treated with tamoxifen in the food for 5 consecutive weeks. Right panels: representative flow cytometry plots, with the gating strategy used for the analysis. Student’s *t* test: ***P *<* 0.001, **P *<* 0.005, *P *<* 0.05. The upper and lower hinges correspond to the first and third quartiles (the 25th and 75th percentiles). The upper whisker extends from the hinge to the highest value that is within 1.5 × IQR of the hinge, where IQR is the inter-quartile range. The lower whisker extends from the hinge to the lowest value within 1.5 × IQR of the hinge. **b** Left panels: Flow cytometry analysis for lymphocytes, macrophages and microglia of the brain of the mice in **a**. Right panels: representative flow cytometry plots, with the gating strategy used for the analysis. **c** Table summarizing the injections performed in the *Ntv-a; LSL-Cas9*; *hUBC-CreERT2* adult mice. Mice injected with RCAS-PDGFB + RCAS-Trp53-gRNA and treated with tamoxifen to induce Cas9 expression, develop high-grade gliomas. **d** H&E and Cas9 IHCs of RCAS-PDGFB + RCAS-Trp53-gRNA tumors for the indicated treatment. High-grade glioma features and Cas9 expression are present only in the tamoxifen-treated mice. Scale bars: H&E 100 μm; IHCs 50 μm

Four-week-old *Ntv-a; LSL-Cas9*; *hUBC-CreERT2*^*+/+*^ and *Ntv-a; LSL-Cas9*; *hUBC-CreERT2*^*+/T*^ mice were treated with tamoxifen-containing diet for a total of 5 weeks. Whole blood samples were taken every 2 weeks. At the end of the experiment (week 9), blood, spleen, and brain tissue were harvested and analyzed by flow cytometry, real-time quantitative PCR and immunofluorescence (Supplementary Fig. 4a). After 5 weeks of tamoxifen treatment we observed high percentage of EGFP-positive cells in the blood (around 50%) and lower number in spleen and brain (approximately 10–15%) (Fig. 3a, b and Supplementary Fig. 4c). Mice did not show any signs of inflammation and splenomegaly was not observed. Circulating levels of T cells, B cells, granulocytes and monocytes were determined by flow cytometry in the blood and spleen. While there were no significant differences neither in T cells (CD3^+^CD4^+^) nor B cells (CD3^−^B220^+^), there was a trend towards decreased circulating Gr-1-positive neutrophils in the blood of the *Ntv-a; LSL-Cas9*; *hUBC-CreERT2*^*+/T*^ tamoxifen-treated mice, which could signify inefficient production of these cells in the bone marrow (Fig. 3a). Despite this reduction, we did not detect significant differences in neither the number nor the percentage of granulocytes in whole blood cell counts (Supplementary Fig. 4b). Flow cytometry in the brain showed no changes in lymphocytes, microglia or macrophages with the gating strategy described previously^35^ using CD45 and CD11b. Furthermore, qPCR analysis indicated no major differences in mRNA expression of a panel of microglia activation-specific markers (*CD45*, *IL12a-1*, *P2ry12*, *Tmem119*, *Cx3cr1* and *Iba-1*) (Supplementary Fig. 4d).

Since our analysis did not suggest an evident immune response to Cas9 expression in the *Ntv-a; LSL-Cas9*; *hUBC-CreERT2*^*+/T*^ mice, we proceeded to inject them with the RCAS-PDGFB/RCAS-gRNA. Four-week-old adult mice were injected intracranially with the RCAS-PDGFB in combination with either RCAS-Trp53-gRNA or RCAS-Ctrl-gRNA. Two weeks after injection, the mice were separated in two groups and treated for two weeks with either mock-treatment or tamoxifen (see Methods for details). Mice were then killed either upon sign of tumor development or at the end of the experiment (90 days). As for the *Ntv-a; Nes-Cre; LSL-Cas9* and *Gtv-a; hGFAP-Cre; LSL-Cas9* strains, none of the *Ntv-a; LSL-Cas9*; *hUBC-CreERT2*^*+/T*^ mice injected with the RCAS-PDGFB and RCAS-Ctrl-gRNA developed tumors (Fig. 3c). While only one out of three of the mock-treated *Ntv-a; LSL-Cas9*; *hUBC-CreERT2*^*+/T*^ mice injected with the RCAS-PDGFB and RCAS-Trp53-gRNA developed a low-grade tumor at 84 days, all the mice treated with tamoxifen were killed at earlier time due to high-grade gliomas (Fig. 3c, d). It is important to note that, since no expression of Cas9 was detected (Fig. 3d, bottom left panel), the low-grade tumor observed in the mock treatment group does not appear to be related to possible leakiness of the CreERT2 system. Although very rarely, the RCAS-PDGFB alone is sufficient to induce tumor formation in adult *Ntv-a* mice^18^. To complement these studies, we then performed a separate set of injections of the RCAS-PDGFB in combination with the RCAS-Trp53-gRNA in a cohort of *Ntv-a; LSL-Cas9* 4-week-old mice, either *hUBC-CreERT2*^*+/+*^ or *hUBC-CreERT2*^*+/T*^. Two weeks after injection, tamoxifen was administered in the diet. Tumor formation was observed exclusively in the *hUBC-CreERT2*^*+/T*^ genetic background (Supplementary Fig. 4e).

### The *Bcan-Ntrk1* gene fusion produces high-grade gliomas

Gene fusions have been documented as cancer drivers for more than three decades, providing valuable insights into the tumorigenesis process. The occurrence and importance of gene fusions in glioma has been appreciated only recently, largely due to high-throughput technologies, and gene fusions have been indicated as one of the major genomic abnormalities in GBM^36^. The functional role of the vast majority of these alterations is completely unexplored. Recurrent gene fusions involving the Trk receptor family (TrkA, B and C coded by *NTRK1*, *2* and *3*, respectively) have been recently described in a variety of tumors, including gliomas^25,37,38^. Here, we decided to focus on the *BCAN-NTRK1* gene fusion, identified in glioblastoma and glioneuronal tumors^39,40^.

**Fig. 4:**
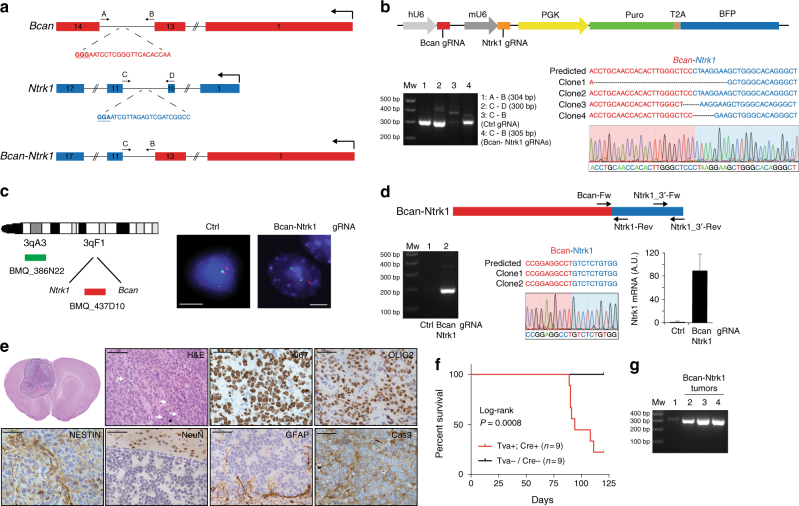
*Bcan-Ntrk1* gene fusion drives high-grade glioma formation. **a** Schematic representation of the *Bcan* and *Ntrk1* gene loci and the *Bcan-Ntrk1* gene fusion. Indicated are the gRNAs targeting both genes and the primers used for the PCR amplification of the indicated genomic regions. **b** Top panel: RCAS-gRNA-pair vector expressing the Bcan and Ntrk1 gRNAs. Bottom panels: PCRs were performed with the specified primers on genomic DNA extracted from the *p53-null* TVA-Cas9 NSCs transduced with the indicated gRNAs. The PCR band for the Bcan-Ntrk1gRNA infected cells was sub-cloned and analyzed by Sanger sequencing. The sequences of four independent clones and a representative chromatogram are shown. **c** Left panel: Diagram of fluorescence in situ hybridization (FISH) probe design. BAC clone BMQ-437D10 (red) is located within the deleted region and BMQ-386N22 (green) is used as a control of chromosome 3. Mouse BACs are represented as green and red bars. Right panels: Representative FISH results using the two-color probe designed to detect the Ntrk1-Bcan intergenic microdeletion. The control green signal was used to count the number of chromosomes 3. The loss of the red signals indicates the microdeletions. Scale bar: 5 μm. **d** Top panel: Schematic representation of *Bcan-Ntrk1* fusion transcript. Bottom panels: (left) RT-PCRs were performed on the mRNA from the *p53-null* TVA-Cas9 NSCs transduced with the indicated gRNAs, using the Bcan-Fw and Ntrk1-Rev primers; (middle) the PCR band was sub-cloned and the sequences of two independent clones and a representative chromatogram are shown; (right) qPCR, with the Ntrk1_3′-Fw and Ntrk1_3′-Rev primers, showing the upregulation of the Ntrk1 mRNA in the cells expressing the *Bcan-Ntrk1* fusion. Data presented as mean ± SD (*n* = 3); A.U. arbitrary unit. **e** H&E and IHCs using the indicated antibodies. White arrows point to mitotic figures. Scale bars: H&E 100 μm; IHCs 50 μm. **f** Kaplan Meier survival curves of RCAS-Bcan-Ntrk1-gRNA pair-induced gliomas generated in *Gtv-a; hGFAP-Cre; LSL-Cas9; p53*^*lox/lox*^ injected mice. Tva^−^ or Cre^−^ control littermates were used as injection controls. Log-rank *P* value = 0.0008. **g** A PCR performed on genomic DNA extracted from Bcan-Ntrk1 tumor tissues (lanes 2–4) confirmed the presence of the *Bcan-Ntrk1* gene fusion. Genomic DNA from NSCs was used as a negative control (lane 1)

*BCAN* and *NTRK1* are located on chromosome (Chr) 1 q23.1 and the *BCAN*-*NTRK1* fusion gene results from an intra-chromosomal deletion that juxtapose the *BCAN* exon 13 with the *NTRK1* exon 11 (Supplementary Fig. 5a). While *BCAN*, that codes for the glycoprotein Brevican, is highly expressed in the adult human brain, *NTRK1* is almost undetectable (Supplementary Fig. 5b). Analogously, the mouse homologs, *Bcan* and *Ntrk1*, are located on the same chromosome (Chr3) and have a similar expression pattern to their human counterparts (Supplementary Fig. 5a-b). To generate the *Bcan*-*Ntrk1* gene fusion we designed gRNAs in the introns 13 and 10 of *Bcan* and *Ntrk1*, respectively (Fig. 4a). The pair of gRNAs was subsequently cloned into an RCAS plasmid containing both a hU6 and mU6 promoters (hU6-gRNA-mU6-gRNA-PGK-Puro-T2A-BFP) (RCAS-gRNA-pair) (Fig. 4b, top panel). The RCAS-gRNA-pair vector was then used to infect NSCs isolated from *Gtv-a; hGFAP-Cre; LSL-Cas9; p53*^*lox/lox*^ pups (*p53-null* TVA-Cas9 NSCs). Generation of the expected chromosomal deletion was tested by PCR on genomic DNA, and later analyzed by Sanger sequencing (Fig. 4b, bottom panel). Fluorescence in situ hybridization (FISH) evidenced that approximately 40% of the RCAS-gRNA-pair-transduced cells (80/209) showed loss of one copy of the probe located between the *Ntrk1* and *Bcan* gene, indicating that the generation of the *Bcan*-*Ntrk1* rearrangement is a relatively efficient process (Fig. 4c). We also confirmed the expression of the *Bcan*-*Ntrk1* fusion transcript by reverse transcription PCR (RT-PCR) and Sanger sequencing of a cDNA fragment overlapping the fusion exon junction (Fig. 4d). Analogously to what has been observed in the GBM patients carrying the *BCAN*-*NTRK1* fusion (Supplementary Fig. 5c), the generation of the *Bcan*-*Ntrk1* rearrangement led to exceptionally high levels of the *Ntrk1* 3′ mRNA region involved in the gene fusion (Fig. 4d, bottom right panel).

To test if the *Bcan*-*Ntrk1* gene fusion was sufficient to drive glioma formation we injected intracranially into *NOD/SCID* mice the *p53-null* TVA-Cas9 NSCs infected with the RCAS-gRNA-pair. While none (0/5) of the mice injected with the control NSCs developed tumors during the observation period (90 days), four out of the six mice injected with the Bcan-Ntrk1gRNA pairs had to be killed due to sign of tumor formation (with mean survival of 72 ± 14 days). Histopathological examination of the tumors evidenced a series of high-grade glioma features: nuclear atypia, high number of mitotic figures, necrotic areas and infiltration in the normal brain parenchyma (Fig. 4e and Supplementary Fig. 5d-e). Bcan-Ntrk1*-*induced tumors showed elevated percentage of Ki67-positive cells, were positive for OLIG2 and NESTIN, negative for the neuronal marker NeuN and GFAP-positive cells were almost exclusively detected at the normal/tumor border (Fig. 4e). The presence of Olig2-positive cells might suggest the resemblance to GBMs with oligodendroglioma component, which represent approximately 20% of primary GBMs^41^. IHC staining with a pan-TRK antibody showed positive signal in Bcan-Ntrk1*-*induced tumors and not in control PDGFB-induced tumor (Supplementary Fig. 5h). Additionally, we confirmed by genomic PCR and FISH analysis the presence of the *Bcan*-*Ntrk1* gene fusion on cells isolated from the tumor-bearing mice, propagated in vitro as tumorspheres (Supplementary Fig. 5g-h). Strikingly, these tumorspheres expressed very high levels of *Ntrk1* as compared to both the NSCs control or to the Bcan-Ntrk1 NSCs prior intracranial injection (Supplementary Fig. 5i).

We then evaluated whether the RCAS-Bcan-Ntrk1 gRNA-pair was able to induce glioma formation when directly delivered in vivo into *Gtv-a; hGFAP-Cre; LSL-Cas9; p53*^*lox/lox*^ pups. Indeed, approximately 80% (7/9) of the injected Tva^+^; Cre^+^ mice developed tumors with a median survival of 94 days (Fig. 4f). The tumors carried the *Bcan*-*Ntrk1* gene fusion (Fig. 4g) and closely resembled at histological level those obtained in the transplantation model (Supplementary Fig. 5j). No tumors were observed in either Tva^−^ or Cre^−^ control littermates.

Lastly, RNA sequencing confirmed the presence of the *Bcan*-*Ntrk1* fusion transcript in tumorspheres derived from both models (NSCs transplantation and RCAS-gRNA pair direct injection) (Supplementary Table 2) and differential expression analysis showed that *Ntrk1* was the most upregulated gene (log2 fold change = 10.01, FDR = 2.13^−50^), as compared to control *p53-null* TVA-Cas9 NSCs (Supplementary Fig. 5k and Supplementary Data 1). Also worthy of note is the significant downregulation of *Ntrk2* (log2 fold change = −1.8, FDR = 0.006), possibly indicating a negative feedback loop that regulates Trk receptors signaling (Supplementary Fig. 5l).

### Bcan-Ntrk1 gliomas are sensitive to entrectinib

**Fig. 5:**
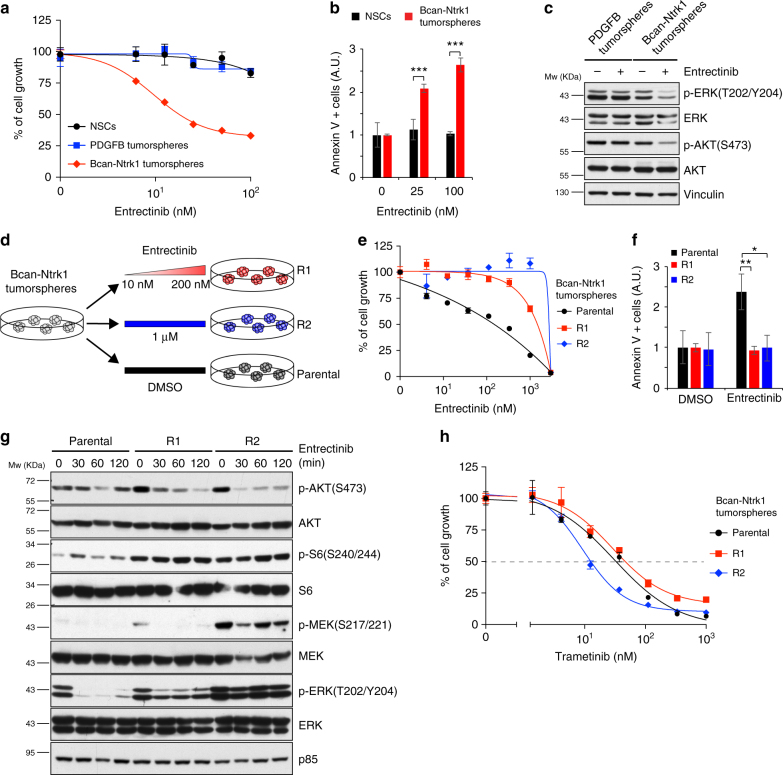
Bcan-Ntrk1-induced gliomas develop resistance to the TRK inhibitor entrectinib. **a** Cell proliferation assay performed on *p53-null* TVA-Cas9 NSCs, Bcan-Ntrk1 or PDGFB tumorspheres exposed for 96 h to increasing doses of entrectinib, a pan-TRK inhibitor. **b** Entrectinib treatment in Bcan-Ntrk1 tumorspheres induces an increase in apoptosis, as measured by Annexin V-positive cells, 48 h after treatment. Data from a representative of two experiments are presented as mean ± SD (*n* = 3); Student’s *t* test: ***P *<* 0.001; A.U. arbitrary unit. **c** Western blot analysis using the specified antibodies on Bcan-Ntrk1 or PDGFB tumorspheres grown for overnight in absence of growth factors and then treated with entrectinib (1 μM) for 16 h. **d** Schematic representation of the generation of entrectinib-resistant cell lines (see text for details). **e** Cell proliferation assay performed on parental Bcan-Ntrk1, R1 and R2 tumorspheres exposed for 96 h to increasing doses of entrectinib. **f** Absence of apoptosis induction in entrectinib-resistant cells, as measured by Annexin V-positive cells, 72 h after entrectinib treatment (100 nM). Data from a representative of two experiments are presented as mean ± SD (*n* = 3); Student’s *t* test: **P *<* 0.005, *P *<* 0.05; A.U. arbitrary unit. **g** Western blot analysis using the specified antibodies on parental Bcan-Ntrk1, R1 and R2 tumorspheres treated with entrectinib (1 μM) for the indicated timepoint. **h** Cell proliferation assay performed on parental Bcan-Ntrk1, R1 and R2 tumorspheres exposed for 96 h to increasing doses of trametinib

There has been a lot of interest lately in targeting *NTRK* gene fusions across multiple tumor types^42^. Entrectinib is a first-in-class pan-TRK kinase inhibitor currently undergoing clinical trials in a variety of cancers. As shown in Fig. 5a, the Bcan-Ntrk1 tumorspheres were exquisitely sensitive to entrectinib, while no effect was observed either on control *p53-null* TVA-Cas9 NSCs or on PDGFB-induced tumorspheres. TRK inhibition led to a significant reduction of tumor cells growth associated with an increase of the number of apoptotic cells (Fig. 5a, b). Western blot analysis confirmed that phosphorylation of TrkA downstream signaling molecules, such as AKT and ERK, was readily reduced by entrectinib in Bcan-Ntrk1 but not in the control PDGFB-tumorspheres (Fig. 5c).

Analogously to what observed for other receptor tyrosine kinase (RTK) inhibitors^43,44^, early clinical trials using entrectinib on patients carrying either *NTRK1* or *NTRK3* rearrangements have evidenced the emergence of drug resistance after marked initial responses^45,46^. Genetic profiling of the tumors and xenopatient samples displayed acquisition of point mutations in the catalytic domain of *NTRK1* (p.G595R and p.G667C) and *NTRK3* (G623R)^45,46^.

To study mechanisms of resistance to TRK inhibition and identify potential therapeutic approaches to overcome such resistance, we generated entrectinib-resistant glioma cell lines. Bcan-Ntrk1 tumorspheres were exposed to either escalating doses (R1, 10–200 nM) or acute constant dose (R2, 1μM) of entrectinib until resistant cells emerged (Fig. 5d). Proliferation and apoptosis assays of R1 and R2 derivatives confirmed the entrectinib resistance of these cells, as compared to the parental Bcan-Ntrk1 tumorspheres (Fig. 5e, f). Sanger sequencing evidenced no mutations in the *Ntrk1* catalytic domain, suggesting that other mechanisms, rather than solvent-front mutations, were responsible for the drug resistance. Biochemical characterization of the entrectinib-resistant cell lines showed increased level of activation of AKT/S6 and MAPK/ERK signaling pathways (Fig. 5g), with sustained activation of the signaling also in the presence of the drug. Due to the particularly high levels of MAPK/ERK activation in the R2 cells, we hypothesized that these cells might have become addicted to MAPK signaling. Indeed, R2 cells showed an evident higher sensitivity to the MEK inhibitor trametinib, as compared both to R1 and parental Bcan-Ntrk1 tumorspheres (Fig. 5h). These data suggest that MEK inhibition might represent a novel approach for entrectinib-resistant tumors.

### Generation of the *Myb-Qk* chromosomal translocation

The human *BCAN-NTRK1* and mouse *Bcan*-*Ntrk1* fusions are generated by a small chromosomal deletion of approximately 200 Kb. To test whether the RCAS-TVA-CRISPR-Cas9 system was also suitable for generating inter-chromosomal translocations, we decided to model the *MYB-QKI* gene fusion, a recently identified putative driver of a subtype of pediatric low-grade gliomas (PLGG), known as angiocentric gliomas^47^. Although *MYB* and *QKI* are both located on Chr6 in human, the mouse homologs *Myb* and *Qk* are located on different chromosomes, Chr10 and Chr17, respectively (Supplementary Fig. 6a).

*MYB* encodes for a transcription factor that is a key regulator of hematopoietic cell proliferation and deregulated MYB activity has been observed in variety of human cancers. *QKI* is a tumor suppressor gene that encodes for a RNA-binding protein; QUAKING, that plays a role in the development of the CNS, among other organs. Several *MYB-QKI* gene fusions have been described in angiocentric gliomas, all of them involved the same *QKI* 3′ region (exons 5−8) fused to different MYB exons (1–9, 1–11 or 1–15)^47^. Here, we focused on the most frequent *MYB* (exons 1–9) *- QKI* (exons 5−8) fusion event.

**Fig. 6:**
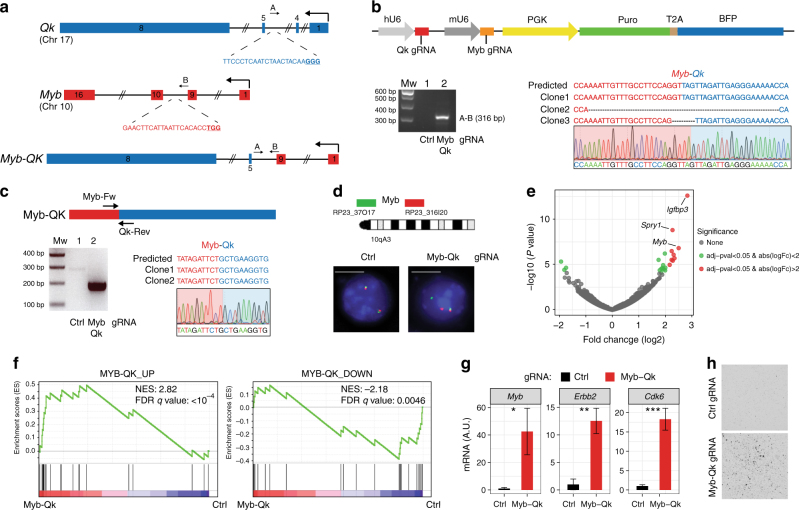
Generation of the *Myb-Qk* chromosomal translocation. **a** Schematic representation of the *Myb* and *Qk* gene loci and the *Myb-Qk* gene fusion. Indicated are the gRNAs targeting both genes and the primers used for the PCR amplification of the indicated genomic regions. **b** Top panel: RCAS-gRNA-pair vector expressing the Myb and Qk gRNAs. Bottom panels: PCRs were performed with the specified primers on genomic DNA extracted from the *p53-null* TVA-Cas9 NSCs transduced with the indicated gRNAs. The PCR band for the Myb-Qk gRNA infected cells was sub-cloned and analyzed by Sanger sequencing. The sequences of three independent clones and a representative chromatogram are shown. **c** Top panel: Schematic representation of *Myb-Qk* fusion transcript. Bottom panels: (left) RT-PCRs were performed on the mRNA from the *p53-null* TVA-Cas9 NSCs transduced with the indicated gRNAs, using the Myb-Fw and Qk-Rev primers; (right) the PCR band was sub-cloned and the sequences of two independent clones and a representative chromatogram are shown. **d** Top panel: Diagram of FISH probe design (see Methods for details). Mouse BACs are represented as green and red bars. Bottom panels: Representative FISH results using the break-a-part probe designed to detect the Myb-Qk translocation. Scale bar: 5 μm. **e** Volcano plot of differential gene expression in *Myb-Qk* (*n* = 2) versus Ctrl gRNA cells (*n* = 2). **f** GSEA analysis using the published MYB-QK signature genes^47^. NES normalized enrichment score, FDR false discovery rate. **g** qPCR analysis shows the upregulation of *Myb-QK*-activated genes in the *p53-null* TVA-Cas9 NSCs expressing the *Myb-Qk* fusion. Data presented as mean ± SD (*n* = 3); Student’s *t* test: ***P *<* 0.001, **P *<* 0.005, *P *<* 0.05; A.U. arbitrary unit. **h** *p53-null* TVA-Cas9 NSCs transduced with the Myb and Qk gRNAs, but not Ctrl gRNA, are able to growth in soft agar

To generate the mouse *Myb* (exons1–9) - *Qk* (exons 5−8) fusion, we designed gRNAs in the intron 4 for *Myb* and 9 for *Qk* (Fig. 6a), and we cloned them into the RCAS-gRNA-pair construct (Fig. 6b, top panel). Genomic PCR and Sanger sequencing from *p53-null* TVA-Cas9 NSCs infected with the RCAS-Qk-Myb gRNA-pair confirmed the generation of the *Myb-Qk* fusion (Fig. 6b, bottom panel). By RT-PCR we also observed the expression of the *Myb-Qk* transcript (Fig. 6c). Furthermore, we used FISH assay using a Myb break-apart (BA) probe to evaluate the frequency of cells carrying the desired t(10;17)/*Myb-Qk* chromosome translocation. Approximately 9% of the cells (6/74) showed broken signals of the BA_FISH flanking the *Myb* gene (Fig. 6d), indicating the generation of the *Myb* rearrangement.

In human and mouse normal adult brain, Myb mRNA expression is almost undetectable (Supplementary Fig. 6b). The *MYB-QKI* fusion has been shown to function as a transcription factor and to activate the *MYB* promoter, possibly contributing to an autoregulatory feedback loop^47^. To functionally validate the *Myb-Qk* gene fusion, we performed RNA sequencing in two independent *p53-null* TVA-Cas9 NSCs lines infected with either the RCAS-Qk-Myb gRNA-pair or Ctrl gRNA. The RNA-Seq analysis confirmed the occurrence of the *Myb-Qk* fusion transcript and also evidenced the expression of the *Qk-Myb* transcript, resulting from the reciprocal t(17;10) chromosome translocation (Supplementary Table 2). We then performed differential expression analysis and we did identify a small subset of significantly differentially regulated genes in *Myb-Qk* versus Ctrl gRNA cells. Among those genes, *Myb* was the second most upregulated (log2 fold change = 2.49, FDR = 0.0007) (Fig. 6e) (Supplementary Data 2). Moreover, Gene Set Enrichment Analysis (GSEA) (Fig. 6f) showed significant enrichment (MYB-QK_UP: NES = 2.82, FDR *q* value < 10^−4^; MYB-QK_down: NES = −2.18, FDR *q* value = 0.0046) for the previously identified *MYB-QKI* -dependent gene signatures^47^. The expression of some of these genes (*Myb, Erbb2*, *Cdk6*) was also further validated by qPCR analysis (Fig. 6g).

We then tested the transforming potential of the *p53-null* NSCs expressing the *Myb-Qk* fusion. Soft-agar assay showed that Myb-Qk cells had an increased colony-forming ability as compared to the Ctrl gRNA cells (Fig. 6h). However, these cells were not able to form tumors in vivo when transplanted intracranially into *NOD/SCID* mice, during a 4-month observation time.

### Modeling BRAF V600E mutation by homology directed repair

One of the known applications of the CRISPR-Cas9 system is to induce point mutations through Homologous Recombination (HR). Delivery of a gRNA with either double-stranded DNA (dsDNA) or single-stranded DNA (ssDNA) repair templates, containing a desired modified sequence together with variable length upstream and downstream homology arms, has been used to recreate oncogenic driver mutations^4,7,8,48^.

Activating mutations in the *BRAF* kinase gene (V600E) have been identified in various types of pediatric gliomas (Pilocytic astrocytomas (<10%), PXAs (WHO grades II and III; 50–65% cases), gangliogliomas (20–75% cases)) and also adult high-grade gliomas (5%)^49^.

**Fig. 7:**
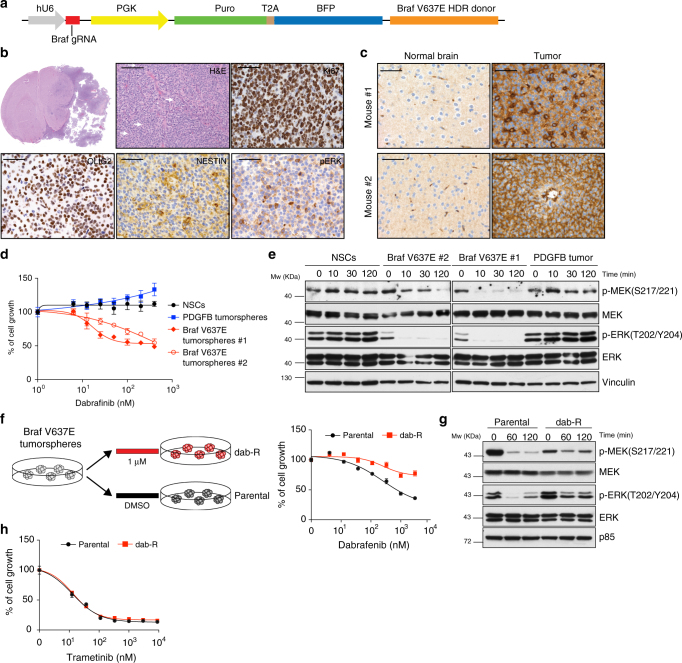
Glioma formation induced by *Braf*^*V637E*^. **a** Schematic representation of the plasmid carrying the Braf gRNA and the Braf^V637E^ Homology-Directed-Repair (HDR) donor. **b** H&E and IHCs using the indicated antibodies. White arrows point to mitotic figures. Scale bars: H&E 100 μm; IHCs 50 μm. **c** IHCs using an anti-BRAF V600E antibody on two Braf^V637E^ mutant tumors. Contralateral normal brain was used as a negative control. **d** Cell proliferation assay performed on *p53-null* TVA-Cas9 NSCs and *Braf*^*V637E*^ tumorspheres exposed for 96 h to increasing doses of dabrafenib. **e** Western blot analysis using the specified antibodies on *p53-null* TVA-Cas9 NSCs and *Braf*^*V637E*^ tumorspheres grown overnight in absence of growth factors and then treated with dabrafenib (200 nM) for the indicated time. **f** Left panel: schematic representation of the generation of dabrafenib-resistant cell line (dab-R). Right panel: cell proliferation assay performed on parental *Braf*^*V637E*^ and dab-R tumorspheres exposed for 96 h to increasing doses of dabrafenib. **g** Western blot analysis using the specified antibodies on parental *Braf*^*V637E*^ and dab-R tumorspheres grown overnight in absence of growth factors and then treated with dabrafenib (200 nM) for the indicated time points. **h** Cell proliferation assay performed on parental *Braf*^*V637E*^ and dab-R tumorspheres exposed for 96 h to increasing doses of trametinib

To model a missense gain-of-function *Braf* mutation we used the strategy previously described to generate a *Kras*^*G12D*^ mutation^4^ and designed a homology-directed repair (HDR) donor template, which comprises of an 800 bp genomic sequence covering exon 18 of the mouse *Braf* gene. This HDR donor encoded: (i) a valin (V) to glutamine (E) mutation in the amino acid position 637 (V637E), resulting in the oncogenic *Braf*^*V637E*^ mutation, homologous to the human *BRAF*^*V600E*^; (ii) 11 synonymous single-nucleotide changes to discriminate the difference between the donor and wild-type sequences and to mutate the protospacer-adjacent motif (PAM) to avoid donor DNA cleavage by Cas9 (Supplementary Data 3). The HDR donor template, together with a gRNA targeting a sequence 22 bp upstream the Braf V637 residue, were subsequently cloned into a lentiviral vector (Fig. 7a) and transduced into the *p53-null* TVA-Cas9 NSCs. We then evaluated the efficiency of HDR-mediated *Braf* mutation by genomic PCR and high-throughput sequencing. The vast majority of the sequence reads (85%) contained either deletions (80%) or insertions (5%) at the gRNA binding site, suggesting that the Cas9 can efficiently cut at this site. Approximately 0.05% of the reads contained the desired V637E mutation and also a second point mutation D624N (Supplementary Fig. 7a). This latter mutation, although undesired, is a conservative mutation from an aspartate to an asparagine residue and it is not expected to have any functional consequence on BRAF activity. The relative small frequency of the mutant allele is consistent with the reported mutation frequency obtained in other CRISPR-Cas9 tumor models^4,7^.

When transplanted intracranially into *NOD/SCID* mice, the *p53-null Braf*^*V637E*^ NSCs induce tumor formation in 100% of the injected mice (6/6), with an average survival of 66 ± 11.5 days. Histopathological examination of the tumors evidenced a number of features characteristic of high-grade gliomas: nuclear atypia, high number of mitotic figures, and necrotic areas (Fig. 7b and Supplementary Fig. 7b). Moreover, we observed clusters of tumor cells infiltrating the normal brain parenchyma, with the vast majority of these cells surrounding tumor vessels (Supplementary Fig. 7b), resembling the vascular co-option observed both in primary and metastatic brain tumors. It is also to note the presence of some giant cells (Supplementary Fig. 7c) and areas of the tumors with epithelioid morphology (Supplementary Fig. 7d).

Immunohistochemical analysis of the BRAF mutant tumors revealed high percentage of Ki67-positive cells, positivity for both OLIG2 and NESTIN and elevated MAPK kinase activity, as evidenced by phospho-ERK IHC (Fig. 7b). Additionally, we were able to confirm the expression of the BRAF V637E mutation using an antibody specifically designed to recognize the human BRAF V600E mutant.

To validate that the BRAF mutant tumors were dependent on an active BRAF signaling pathway, we isolated tumorspheres from two of those tumors and we treated them in vitro with dabrafenib, a specific BRAF inhibitor that is currently in clinical trials for *BRAF*^*V600E*^ mutant melanomas. Both tumorspheres lines carried the *Braf*^*V637E*^ mutation as confirmed by PCR and Sanger sequencing (Supplementary Fig. 7e). As shown in Fig. 7d, dabrafenib treatment induced growth reduction in both *Braf*^*V637E*^ tumorspheres, but not in *p53-null* NSCs or PDGFB tumorspheres. Moreover, *NOD/SCID* mice intracranially injected with *Braf*^*V637E*^ tumor cells showed a moderate, although not significant, prolonged survival when treated in vivo with dabrafenib (Supplementary Fig. 7f).

Western blot analysis showed a reduction of MAPK kinase signaling pathway after exposure to dabrafenib in *Braf*^*V637E*^ tumor cells but not in control *p53-null* NSCs or PDGFB tumorspheres (Fig. 7e), thus confirming the dependency on BRAF activation for the maintenance of MEK/ERK signaling in *Braf*^*V637E*^ tumors.

Second-generation BRAF inhibitors, such as dabrafenib and vemurafenib, have produced outstanding improvement in the survival of *BRAF*^*V600E*^ mutant melanomas; however, their efficacy has been limited by acquired resistance to these drugs^50^. To explore the utility of the *Braf*^*V637E*^ tumor model for the study of resistance mechanisms, we generated dabrafenib-resistant cells (dab-R) by exposure of *Braf*^*V637E*^ tumorspheres to an acute constant dose (1 μM) of the drug (Fig. 7f, left panel). Proliferation assays confirmed the dabrafenib resistance of the dab-R cells, as compared to the parental *Braf*^*V637E*^ tumorspheres (Fig. 7f, right panel). Western blot analysis revealed higher phospho-ERK levels, with a concomitant reduction of phospho-MEK, in unperturbed dab-R versus parental cells (Fig. 7g). This observation is consistent with the previously described negative feedback loop, in which ERK phosphorylates and inhibits members of the RAS/RAF/MEK signaling pathway^51^. Most importantly, dab-R cells maintained a sustained activation of the MEK/ERK signaling upon exposure to dabrafenib, in comparison to the parental *Braf*^*V637E*^ tumorspheres (Fig. 7g).

Combination of the BRAF inhibitor dabrafenib with the MEK inhibitor trametinib has been shown to lead to a further improved overall survival in metastatic melanoma carrying the BRAF V600E mutation, as compared with dabrafenib monotherapy^52^. Indeed, dab-R cells showed similar sensitivity to trametinib as compared to the parental *Braf*^*V637E*^ tumorspheres (Fig. 7h). These data would suggest that also in *BRAF*^*V600E*^ mutant gliomas dabrafenib resistance might be mediated by increased activation of the MEK/ERK signaling pathway and that it could be overcome by combining BRAF and MEK inhibitors.

## Discussion

The latest improvements in the genetically engineered mouse modeling have contributed to the understanding of the molecular pathways responsible for tumor initiation and progression. Few elements should be taken in consideration to properly mimic the natural history of a tumor: (a) introduction of the same mutations found in human tumors, ideally in their endogenous loci; (b) the genetic alterations should be silent during embryonic and early postnatal development (with the exception of models of familiar or pediatric tumors); (c) the mutant genes should be expressed in particular target tissues or in specific cell types and (e) the mutations should be present in a limited number of cells. The RCAS-TVA-CRISPR-Cas9 system described here gives the possibility to recapitulate all of these characteristics in one single model.

A number of different knockin TVA mouse models have been published in the past and they have been used to study a variety of cancers: gliomas, medulloblastomas, melanoma, breast, pancreatic, ovarian, and liver cancer^12,53^. Breeding of any of the TVA lines to the knockin Cas9 strain would allow to generate novel somatic genome editing models to study a plethora of tumor types. As a proof-of-principle, we used two different CNS-specific TVA-transgenic lines (*Nestin-tv-a* and *GFAP-tv-a*) to perform functional characterization of various genetic alterations described in human gliomas: (1) knockout of a panel of TSGs recurrently lost or mutated in GBMs (*TP53*, *CDKN2A* and *PTEN*), (2) genomic rearrangements identified in different subtypes of gliomas (*BCAN*-*NTRK1* and *MYB*-*QKI*) and (3) a point mutation (BRAF^*V600E*^) present in a variety of pediatric and adult gliomas.

For the TSGs we selected *Trp53*, *Cdkn2a* and *Pten*, previously shown to cooperate with PDGFB overexpression to induce high-grade gliomas^18,28^. Indeed, co-injection of gRNAs targeting those genes led to the formation of GBMs with high frequency. These data would suggest that combining the expression of specific oncogene drivers with gRNAs for a TSG of interest would quickly provide information on its contribution to the tumorigenesis process. Moreover, by using the *hUBC-CreERT2*^*+/T*^ or other Cre-inducible strains it will be possible to exploit the RCAS-TVA-CRISPR-Cas9 system not only to study tumor initiation, but also tumor progression and maintenance.

Due to the quite recent advancement in the CRISPR-Cas9 technology, very few mouse models have been previously developed to study brain tumorigenesis^6,54,55^. By in utero electroporation (IUE) of the forebrain of mouse embryos using plasmids encoding Cas9 in combination with gRNAs targeting *Nf1*, *Trp53* and *Pten*, Zuckerman and Chen were able to induce highly aggressive tumors that had histopathological features of human GBMs. More recently, Cook and colleagues have used adenoviral (Ad) vectors to express Cas9 and to generate the *Bcan-Ntrk1* rearrangement in the brain of adult mice^55^.

In our opinion there are at least two key issues with the use of IUE and Ad for glioma CRISPR-Cas9 modeling: timing of the gRNA delivery and lack of specificity of the targeted cells. Electroporation is normally performed at E14.5 or E15.5 and genetic alterations at this gestational stage might not be necessarily reflecting the biology of gliomas in the adult. The second issue is that the expression of the Cas9 enzyme from a constitutive promoter, as it has been used in both IUE and Ad studies, does not allow genome editing in a cell-type-specific manner hence not restricting the genetic alteration to the putative cells of origin of gliomas. This latter point is particularly relevant for proper cancer modeling, since it has been shown that the same driver mutations can lead to phenotypically and molecularly diverse glioma subtypes from different pools of adult CNS progenitor cells^56,57^.

The CRISPR-Cas9 system has been previously used to model genomic rearrangements both in human and mouse cells^9–11,55^. Fusion transcripts can generally lead to at least four different situations: (a) increased overexpression of an oncogene (e.g. *IgH-MYC* in leukemia), (b) deregulation of a tumor suppressor gene (e.g. *CHEK2-PP2R2A* in childhood teratoma), (c) generation of a new aberrant protein (e.g. *BCR-ABL1* in leukemia), and (d) a combination of various of the above (e.g. *MYB-QKI* in angiocentric glioma)^47,58^. We have observed that, in human glioma patients and in our mouse model, the *NTRK1* gene fusions led to overexpression of the chimeric *NTRK1* transcripts (Supplementary Fig. 5 panels c, i, and k). Most likely, the very pronounced levels of TrkA kinase activity achieved by the high levels of the chimeric *NTRK1* transcripts are responsible for the oncogenic activity of those fusions. Indeed, we observed that the *Bcan*-*Ntrk1* tumors were finely sensitive to the pan-TRK inhibitor entrectinib.

Acquired resistance, due to point mutations that disrupt the binding between the drug and the kinase domain, has emerged during early clinical trial studies as a possible limitation to the use of entrectinib as a single agent^45,46^. By generating entrectinib-resistant tumorsphere lines, we observed an upregulation of the AKT and MAPK signaling, yet in the absence of the previously reported *NTRK1* mutations p.G595R and p.G667C^45,46^. Although further work will be required to pinpoint the precise mechanisms that lead to an increased MAPK pathway activation, we have presented pre-clinical evidence that MEK inhibitors, such as trametinib, could represent a potential therapeutic strategy to overcome entrectinib resistance.

The *MYB-QKI* rearrangement has been shown to drive tumorigenesis through a tripartite mechanism: MYB activation by truncation, aberrant MYB-QKI expression and hemizygous loss of the tumor suppressor *QKI*^47^. Using the RCAS-TVA-CRISPR-Cas9 system we successfully generated a functional *Myb*-*Qk* gene fusion in mouse cells and we observed an increase in *Myb* activation, as well as differential expression of MYB-QKI-regulated genes (Fig. 6e−g). Differently to what had previously been described for cells in which the MYB-QKI fusion protein was exogenously overexpressed^47^, cells carrying the *Myb*-*Qk* fusion gene had a limited in vivo tumorigenic potential and were not able to grow when intracranially transplanted in *NOD/SCID* mice. A possible explanation to the lack of tumorigenicity is that in our studies we used a mix population of cells in which approximately only 10% of the cells expressed the *Myb*-*Qk* fusion gene. Alternatively, it is conceivable that the *Myb-Qk* level of expression obtained in our experimental model might not be sufficiently high to achieve a full transforming potential, as compared to the exogenous overexpression of the MYB-QKI fusion protein.

Despite that the generation of point mutations with the CRISPR-Cas9 system might represent one of the most powerful feature of this genome editing technology, it is also the most challenging and very few cancer models have been developed with it^4,7,8,48^. Here we generated the first CRISPR-Cas9 model for the BRAF V600E mutation.

The mouse tumors carrying the *Braf*^*V637E*^ knockin mutation (homologous to the human *BRAF*^V600E^ mutant allele) presented certain characteristics that resemble the epithelioid variant of glioblastoma (E-GBM), with some of the tumor cells exhibiting epithelioid traits and discohesiveness. BRAF V600E mutation has been identified in approximately 60% of pleomorphic xanthoastrocytomas (PXAs) and E-GBMs are thought to arise from the malignant transformation of PXAs^59^. Here we have shown that *Braf*^*V637E*^ tumors are quite sensitive to dabrafenib treatment, suggesting that this inhibitor might represent a possible therapeutic approach for those glioma types. Moreover, we have shown that acquired dabrafenib resistance could be overcome by the MEK inhibitor trametinib. Our observations are consistent with the currently tested combination treatment of BRAF and MEK inhibitors in different BRAF V600E mutant gliomas types, including PXAs^60^.

In conclusion, we have developed an extremely powerful and versatile mouse model that combines the somatic genome transfer ability of the RCAS-TVA system with the CRISPR-Cas9 genome editing technology. We believe that such a flexible model will greatly expedite the generation of precise cancer models, therefore accelerating the pre-clinical testing of novel targeted therapies.

## Methods

### DNA constructs and design of guide RNAs

The pKLV-U6gRNA-PGKpuro2ABFP was a gift from Kosuke Yusa (Addgene plasmid # 50946); lentiCas9-Blast was a gift from Feng Zhang (Addgene plasmid # 52962) and pMSCVhygro-Cre was a gift from Kai Ge (Addgene plasmid # 34565). The retroviral RCAS Gateway Destination Vector (RCAS-Y-DV)^61^ was kindly provided by Eric Holland. To sub-clone the gRNA into the RCAS-Y-DV vector we generated a pDONR-gRNA plasmid, by a multiple steps process. First, the region containing the hU6 promoter, *Bbs*I cloning sites, gRNA scaffold, PGK promoter, and selectable markers Puromycin and BFP (Blue Fluorescent protein) (hU6-gRNA-PGK-Puro-T2A-BFP) was amplified by PCR from the pKLV-U6gRNA-PGKpuro2ABFP plasmid using the Platinum Pfx Kit (Invitrogen, Cat. 11708-013) and the primers aTTB Fw and aTTB Rv (Supplementary Table 3). The PCR-amplified product was transferred by site-specific recombination (Gateway BP Clonase, Invitrogen, Cat. 11789-020) into the pDONR221 Vector (Invitrogen, Cat. 12536017) following the manufacturer’s instructions. Lastly, the *Bbs*I restriction site at position 437 was removed by site-directed mutagenesis (QuikChange Lightning Site-Directed Mutagenesis kit, Agilent, Cat. 210518) using the primers pDONR_BbsI_mut-Fw and Rv (Supplementary Table 3). This step was necessary to remove from the pDONR221, a *Bbs*I restriction site outside the gRNA cloning site.

To generate the pDONR-gRNA that expressed also the PDGFB-HA, we performed a PCR using the Platinum Pfx Kit and the primers pDONR-Fw and Rv for the backbone and PDGFB-Fw and Rv for the insert (Supplementary Table 3). The two fragments were then assembled using the Gibson Assembly Master Mix (New England Biolabs, Cat. E2611L). To obtain the final plasmid, the *Bbs*I restriction site in the PDGFB sequence (pDONR-sgRNA-PDGFB position 2822) was removed by site-directed mutagenesis using the primers PDGFB_ BbsI_mut-Fw and Rv.

The pDONR-gRNA plasmids were recombined into the RCAS-Y-DV using the Gateway LR Clonase II Enzyme mix (Invitrogen, Cat. 11791100), following the manufacturer’s instructions. All the constructs were verified by Sanger sequencing.

The gRNA sequences targeting *Cdkn2a*, *Pten* and *Tp53* were previously described^6,23,24^. *Bcan*, *Ntrk1*, *Myb*, *Qk* and *Braf* gRNAs were designed using the Genetic Perturbation Platform web portal (http://portals.broadinstitute.org/gpp/public/analysis-tools/gRNA-design).

For cloning of single gRNAs, oligonucleotides containing the *Bbs*I site and the specific gRNA sequences were annealed, phosphorylated and ligated either into the pDONR-gRNA or the pKLV-U6gRNA(BbsI)-PGKpuro2ABFP previously digested with *Bbs*I. The cloning of the paired gRNA was done according to the protocol described by Vidigal and colleagues^62^. Briefly, the oligonucleotides containing the different gRNA-pairs (Supplementary Table 3) were amplified with Phusion High-Fidelity polymerase (New England Biolabs, M0530S) using primer F5 and R1 (Supplementary Table 3). PCR products were gel-purified and ligated to *Bbs*I-digested pDonor_mU6 plasmid (kindly provided by A. Ventura) by using the Gibson Assembly Master Mix (New England Biolabs, Cat. E2611L). The Gibson reaction was then digested with *Bbs*I at 37 °C for 3 h. The linearized fragment containing the pair gRNA, the mU6 promoter and the gRNA scaffold was gel-purified and cloned into the pDONR-gRNA and then into the RCAS-Y-DV.

The Braf V637E HDR donor (Supplementary Data 3) was synthesized using the GeneArt service from ThermoFisher Scientific and subsequently cloned into the *Pac*I restriction site into the pKLV-U6-Braf gRNA-PGKpuro2ABFP.

### Cell lines

The mouse embryo fibroblast NIH-3T3-TVA cells, kindly provided by Eric Holland, were cultured in DMEM (Sigma-Aldrich, Cat. D5796) + 10% CS (Sigma-Aldrich, Cat. C8056). The Gp2-293 packaging cell line (Clontech, Cat. 631458) were grown in DMEM (Sigma-Aldrich, Cat. D5796) + 10% FBS (Sigma-Aldrich, Cat. F7524). DF1 chicken fibroblasts (ATCC, Cat. CRL-12203) were grown at 39 °C in DMEM containing GlutaMAX-I (Gibco, Cat. 31966-021) and 10% FBS (Sigma-Aldrich, Cat. F7524). The mouse neuronal stem cells (NSCs) used to test gRNA in vitro and in vivo were derived from the whole brain of newborn mice of *Ntv-a; LSL-Cas9* and *Gtv-a; hGFAP-Cre; LSL-Cas9; p53*^*lox/lox*^, respectively. NSCs and tumorspheres were grown in Mouse NeuroCult proliferation kit (Stem Cell Technologies, Cat. 05702), supplemented with 10 ng/ml recombinant human EGF (Gibco, Cat. PHG0313), 20 ng/ml basic-FGF (Sigma-Aldrich, Cat. F0291-25UG), and 1 mg/ml Heparin (Stem Cell Technologies, Cat. 07980).

*Ntv-a; LSL-Cas9* and NIH-3T3-TVA cells were subsequently infected with either the pMSCVhygro-CRE or lentiCas9-Blast, respectively, to induce Cas9 expression.

DF1 cells were transfected with the different RCAS-gRNA retroviral plasmids using FuGENE 6 Transfection reagent (Promega, Cat. E2691), accordingly to manufacturer’s protocol. DF1 RCAS-virus containing media was used to infect NSCs and NIH-3T3-TVA-Cas9. NSCs were infected with four cycles of spin infection (200 × *g* for 2 h) and then selected with 1 μg/ml Puromycin (Sigma-Aldrich, Cat. P8833-25MG).

Viruses, other than RCAS, were generated in Gp2-293 using calcium-phosphate precipitate transfection: lentiviruses (pKLV-U6gRNA-PGKpuro2ABFP and lentiCas9-Blast) were produced by co-transfection with second-generation packaging vectors (pMD2G and psPAX2) and retroviruses (pMSCVhygro-CRE) with VSVg packaging vector. High-titer virus was collected at 36 and 60 h following transfection and used to infect cells in presence of 7 μg/ml polybrene (Sigma-Aldrich, Cat. H9268-5G) for 12 h. Transduced cells were selected after 48 h from the last infection with Blasticidin (3 μg/ml) (Gibco, Cat. A11139-03) or Hygromycin (300 μg/ml) (Sigma-Aldrich, Cat. H3274-25MG).

Entrectinib (RXDX-101), trametinib (GSK1120212), dabrafenib (GSK2118436) were purchased from Selleckchem (Cat. S7998, S2673, S2807, respectively).

### NSCs and tumorspheres preparation

For the derivation of mouse NSCs and tumor-derived neurospheres, the tissue was enzymatically digested with 5 ml of papain digestion solution (0.94 mg/ml papain (Worthington, Cat. LS003119), 0.48 mM EDTA, 0.18 mg/ml *N*-acetyl-l-cysteine (Sigma-Aldrich, Cat. A9165-5G) in Earl’s Balanced Salt Solution (Gibco, Cat. 14155-08)) and incubated at 37 °C for 8 min. After digestion, the enzyme was inactivated by the addition of 2 ml of 0.71 mg/ml ovomucoid (Worthington, Cat. LS003087) and 0.06 mg/ml DNaseI (Sigma-Aldrich, Cat. 10104159001) diluted in Mouse Neurocult NSC basal medium (Stem Cell Technologies, Cat. 05700) without growth factors. The cell suspension was then passed through a 40 μm mesh filter to remove undigested tissue, washed first with PBS and then with 3 ml of ACK lysing buffer (Gibco, Cat. A1049201). Single cells suspension was then centrifuged at a low speed and resuspended in Mouse NeuroCult proliferation kit (Stem Cell Technologies, Cat. 05702), supplemented with 10 ng/ml recombinant human EGF (Gibco, Cat. PHG0313), 20 ng/ml basic-FGF (Sigma-Aldrich, Cat. F0291-25UG), and 1 mg/ml Heparin (Stem Cell Technologies, Cat. 07980).

### Free floating immunofluorescence (FF-IF)

Adult (4−6 weeks) and pups (1 day) brains were fixed with PFA 4% (Electron Microscopy Sciences, Cat. 15713) and then incubated with sucrose 15 and 30%. Each step was done overnight at 4 °C. Brains were then sectioned by using a sliding microtome with freezing stage (Fisher). Sections of 80 μm were blocked in goat serum 10%, BSA 2%, Triton 0.25% and mouse on mouse (MOM) blocking reagent (Vector Laboratories, Cat. BMK-2202) in PBS for 2 h at room temperature (RT). Primary antibodies were incubated overnight at 4 °C in the blocking solution and the following day for 30 min at RT as detailed: GFAP (Millipore, MAB360, 1:500), NESTIN (BD Pharmingen, #556309, 1:100) and EGFP (Aves Labs, GFP-1010, 1:1000). Slices were then washed in PBS-Triton 0.25% and incubated with the secondary antibody for 2 h. Secondary antibodies were from Invitrogen (Alexa-Fluor anti-chicken-488, anti-rabbit-555, anti-mouse-555). After extensive washing in PBS-Triton 0.25%, nuclei were stained with DAPI for 3 min at RT. Sections were mounted with ProLong Gold Antifade reagent (Invitrogen, Cat. P10144).

Brain mapping was performed with a TCS SP5 confocal microscope (Leica Microsystems) equipped with Leica HCS-A and custom-made iMSRC software^63^. Final images were acquired with a ×20 0.7 N.A. dry objective. The regions of interest definition were done on mosaics of the full brain sections acquired with a ×10 0.4 N.A. dry objective.

### Immunoblotting

Cell pellets were lysed with JS lysis buffer (50 mM HEPES, 150 mM NaCl, 1% Glycerol, 1% Triton X-100, 1.5 mM MgCl_2_, 5 mM EGTA) and protein concentrations were determined by DC protein assay kit II (Biorad, Cat. 5000112). Proteins were separated on house-made SDS-PAGE gels and transferred to nitrocellulose membrane (Amersham). Membranes were incubated in blocking buffer (5% milk 0.1% Tween, 10 mM Tris at pH 7.6, 100 mM NaCl) and then with primary antibody either 1 h at room temperature or overnight at 4 °C according to the antibody. Antibodies used for western blot are: p53 (Cell Signaling Technology, #2524, 1:1000), PTEN (Cell Signaling Technology, #9188, 1:1000), p19ARF (Abcam, ab80-100, 1:500), p-AKT (S473) (Cell Signaling Technology, #4060S, 1:1000/2000), AKT (Cell Signaling Technology, #9272S, 1:1000), p-S6 (S240/244), (Cell Signaling Technology, #2215S, 1:1000), S6 (Cell Signaling Technology, #2317, 1:1000), p21 (BD Pharmingen, #556430, 1:1000), p85 (Millipore, #0619, 1:1000), p-ERK(T202/Y204) (Cell Signaling Technology, #9101, 1:2000/3000), ERK (Cell Signaling Technology, #9102, 1:1000), p-MEK(S217/221) (Cell Signaling Technology, #9154, 1:500/1000), MEK (Cell Signaling Technology, #9122 1:1000) and VINCULIN (Sigma-Aldrich, V9131, 1:10000). Anti-mouse or rabbit-HRP-conjugated antibodies (Jackson Immunoresearch) were used to detect desired protein by chemiluminescence with ECL (Amersham, RPN2106). Uncropped scans of the western blots are reported in Supplementary Figures 8–9.

### Immunohistochemistry

Tissue samples were fixed in 10% formalin, paraffin-embedded and cut in 3 μm sections, which were mounted in superfrostplus microscope slides and dried. Tissues were deparaffinized in xylene and re-hydrated through a series of graded ethanol until water. For histopathological analysis, sections were stained with hematoxylin and eosin (H&E). For immunohistochemistry, paraffin sections underwent first antigenic exposure process, endogenous peroxidase was blocked and the slides were then incubated in blocking solution (2.5% BSA, 10% goat serum, with or without MOM IgG, according to the species of primary antibody, in PBS). Incubation with the appropriate primary antibodies was carried out overnight as detailed: GFAP (Millipore MAB360, 1: 500), NeuN (Millipore, MAB377, 1:100), OLIG2 (Millipore, AB9610, 1:400), NESTIN (BD Pharmingen, #556309, 1:100), PTEN (Cell Signaling Technology, #9559, 1:100), p19ARF (Santa Cruz Biotechnology, sc-32748, 1:100), p21 (CNIO monoclonal antibody core, clone 291H/B5, 1:10), cleaved caspase-3 (Asp175) (Cell Signaling Technology, #9661, 1:750). After incubating with the primary antibody, all slides were incubated with appropriate secondary antibodies and the visualization system AB solution (AB solution-Vector, Ref. PK-6100). Finally, slides were dehydrated, cleared and mounted with a permanent mounting medium. The immunohistochemistry for p53 (CNIO monoclonal antibody core, clone POE316A, 1:100), Ki67 (Master Diagnostica, #0003110QD, undiluted), Cas9 (Cell Signaling Technology, #14697, 1:100), pan-TRK (Cell Signaling Technology, #92991, 1:100), p-S6 (S240/244) (Cell Signaling Technology, #92991, 1:500), p-AKT (S473) (Abcam, # ab81283, 1:175) and p16 (CNIO monoclonal antibody core, clone AM-33B, ready to use) was performed using an automated immunostaining platform (Ventana discovery XT, Roche). BRAF^V600E^ immunostaining was performed on a Leica Bond-III stainer (Leica Biosystem, Newcastle, UK) with a 1:100 dilution of anti-BRAF^V600E^ (VE1) mouse monoclonal primary antibody (Spring Bioscience, Pleasanton, CA).

### Blood counts and flow cytometry

For the analysis of the Cas9-induced immune response, 4-week-old *Ntv-a; LSL-Cas9*; *hUBC-CreERT2* mice were fed ad libitum with tamoxifen containing diet for the duration of the experiment (Supplementary Fig. 3a).

Complete blood counts were carried out using the Abacus Junior Vet (Diatron). Cells were isolated from spleen and brain by mechanical disruption. Red blood cells were lysed using the red blood cell lysis buffer (Sigma-Aldrich). All cells were stained with CD45-PerCP (Biolegend, #103130, 1:200), CD3-AF700 (eBiosciences, #56-0032, 1:100), CD4-PECy7 (BD Pharmingen, #552775, 1:200), Gr-1-PE (BD Pharmingen, #553128, 1:200), CD11b-PerCPCy5.5 (BD Pharmingen, #550993, 1:30) and B-220-APC-CY7 (BD Pharmingen, #552094, 1:200). Samples were acquired in an LSR Fortessa (BD, San Jose, CA) equipped with 355, 488, 561 and 640 nm lines. We used pulse processing to exclude cell aggregates and DAPI to exclude dead cells. All data were analyzed using FlowJo 9.9.4 (Treestar, Oregon).

### Cell proliferation and Annexin V staining

NSCs and tumorspheres cells were seeded in 96-well culture plates (4000 per well) in quintuplicate and treated for 96 h. At the end of the incubation period, survival of cells was determined by the MTT assay. Briefly, MTT (Sigma-Aldrich, Cat. M5655) was added to each well and samples were incubated for 4 h before lysing in formazan dissolving solution. Colorimetric intensity was quantified using an ELISA reader at 590 nm. Values were obtained after subtraction of matched blanks (medium only). The OD values of DMSO controls were taken as 100% and values for drug treatment are expressed as % of control.

The soft-agar growth assay was performed by seeding cells in triplicates at 300,000 cells/well in NSCs culture medium containing 0.4% Noble agar (Sigma-Aldrich, Cat. A5431). Cells were plated on top of a layer of NSCs culture medium containing 0.65% Nobel agar. Colonies were stained 3 weeks after plating with 2 mg/ml of thiazolyl blue tetrazolim bromide (Sigma-Aldrich, Cat. M5655) for 1 h at 37 °C.

Annexin V staining was performed using an APC-Annexin V antibody, according to the manufacture procedure (BD Pharmingen, Cat 550474).

### Reverse transcription quantitative PCR

RNA from NSCs and frozen tissue was isolated with TRIzol reagent (Invitrogen, Cat. 15596-026) according to the manufacturer’s instructions. For reverse transcription PCR (RT-PCR), 500 ng of total RNA was reverse transcribed using the High Capacity cDNA Reverse Transcription Kit (Applied Biosystems, Cat. 4368814). The cDNA was used either for quantitative PCR or Sanger sequencing. Quantitative PCR was performed using the SYBR-Select Master Mix (Applied Biosystems, Cat. 4472908) according to the manufacturer’s instructions. qPCRs were run and the melting curves of the amplified products were used to determine the specificity of the amplification. The threshold cycle number for the genes analyzed was normalized to GAPDH. Sequences of the primers used are listed in Supplementary Table 3.

For Sanger sequencing PCR fragments, cDNA was PCR-amplified using primers listed in Supplementary Table 3, in-gel purified and ligated into the pGEM-T Easy vector (Promega, Cat. A1360) and submitted to sequence.

### Genomic DNA isolation and analysis

Genomic DNA was isolated by proteinase K/sodium dodecyl sulfate (SDS)/phenol extraction method described briefly below. Cell pellets were incubated in lysis buffer (10 mM Tris-HCl pH 8, 100 mM NaCl, 0.5 mM EDTA, 10% SDS and proteinase K) for 4 h at 55 °C. Samples were extracted using phenol:chloroform (1:1) and Phase Lock heavy 2 ml tubes (5PRIME, Cat. 2302830). The aqueous phase was recovered to fresh tubes and 0.1 M sodium acetate and 100% cold ethanol were added. Samples were centrifuged at 20,000 × *g* for 25 min. After washing in 70% cold ethanol, draining and dissolving in water, genomic DNA was quantified. One hundred nanograms of DNA was amplified with specific primers listed in Supplementary Table 3. PCR products were cloned into the pGEM-T Easy vector and submitted for sequencing.

### Fluorescence in situ hybridization

Two sets of FISH probes were used to study the deletion between the *Ntrk1* and *Bcan* mouse genes. BMQ-437D10 bacterial artificial chromosome (BAC) that map at the intergenic *Ntrk1-Bcan* (3qF1 cytoband) was purchased from Source Bioscience and labeled by Nick translation assay with Texas Red fluorochrome to generate a locus-specific FISH probe. BMQ-386N22 BAC clone (3qA3 cytoband) was labeled with Spectrum Green fluorochrome to generate a control probe to enumerate mouse chromosome 3. On the other hand, two sets of FISH probes were used to study the Myb mouse gene rearrangement. RP23-37O17 and RP23-316I20 BACs that map at the 5′ and 3′ *Myb* flanking regions (10qA3 cytoband) were purchased from BACPAC Resoirce CHORI and labeled by Nick translation assay with Spectrum Green and Spectrum Orange fluorochromes, respectively, to generate a break-apart locus-specific FISH probe. FISH analyses were performed according to the manufacturers’ instructions, as previously described^64^, on Carnoy’s fixed cells mounted on positively charged slides (SuperFrost, Thermo Scientific). Briefly, the slides were first dehydrated followed by a denaturing step in the presence of the FISH probe at 85 °C for 10 min and left overnight for hybridization at 45 °C in a DAKO hybridizer machine. Finally, the slides were washed with 20× SSC (saline-sodium citrate) buffer with detergent Tween-20 at 63 °C, and mounted in fluorescence mounting medium (DAPI). FISH signals were manually enumerated within nuclei. FISH images were also captured using a CCD camera (Photometrics SenSys camera) connected to a PC running the Zytovision image analysis system (Applied Imaging Ltd., UK) with focus motor and Z stack software.

### RNA sequencing and analysis

Eight hundred nanograms of total RNA from the samples was used. PolyA+ fraction was purified and randomly fragmented, converted to double-stranded cDNA and processed through subsequent enzymatic treatments of end-repair, dA-tailing, and ligation to adapters as in Illumina’s “TruSeq Stranded mRNA LT Sample Prep Kit” (this kit incorporates dUTP during second strand cDNA synthesis, which implies that only the cDNA strand generated during first strand synthesis is eventually sequenced). Adapter-ligated library was completed by PCR with Illumina PE primers. The resulting purified cDNA library was applied to an Illumina flow cell for cluster generation and sequenced on an Illumina NovaSeq 6000 by following manufacturer’s protocols. 101 base paired-end sequencing reads were analyzed with the next*presso* pipeline^65^, as follows: sequencing quality was checked with FastQC v0.11.0 (http://www.bioinformatics.babraham.ac.uk/projects/fastqc/); reads were aligned to the mouse genome (NCBI37/mm9) with TopHat-2.0.10^66^ using Bowtie 1.0.0^67^ and SAMtools 0.1.19^68^, allowing 3 mismatches and 20 multihits; transcripts quantification and differential expression were calculated with Cufflinks 2.2.1^66^, using the mouse NCBI37/mm9 transcript annotations from https://ccb.jhu.edu/software/tophat/igenomes.shtml. GSEAPreranked^69^ was used to perform gene set enrichment analysis of the described gene signatures on a pre-ranked gene list, setting 1000 gene set permutations. Fusion transcripts were identified with TophatFusion^70^.

GBM expression subtypes were determined with ssGSEA using the ‘ssgsea.GBM.classification’ R package^30^.

### Analysis of Braf mutant allele frequency

Mouse Braf genomic locus was amplified using the primers P7Fw-Idx20 and P5Rev (Supplementary Table 3). The resulting PCR product was purified by spin-column clean-up and sequenced, using the Braf_seq oligo (Supplementary Table 3), on an Illumina platform by following manufacturer’s protocols. Fastq reads files were analyzed with the CRISPResso web application (http://www.crispresso.rocks)^71^.

### Mouse strains and husbandry

*Nestin-tv-a* and *GFAP-tv-a*^13,14^ were generously provided by Eric Holland. *Rosa26-LSL-Cas9* knockin mouse strain^4^ was purchased from The Jackson Laboratory (Cat. 024857). *Nestin-Cre*^22^, *hGFAP-Cre*^21^, *hUBC-CreERT2*^31^ transgenic lines were kindly provided by various researchers at the Spanish National Cancer Research Centre (Marcos Malumbres, Mariano Barbacid and Maria Blasco). Mice were housed in the specific pathogen-free animal house of the Spanish National Cancer Research Centre under conditions in accordance with the recommendations of the Federation of European Laboratory Animal Science Associations (FELASA). All animal experiments were approved by the Ethical Committee (CEIyBA) and performed in accordance with the guidelines stated in the International Guiding Principles for Biomedical Research Involving Animals, developed by the Council for International Organizations of Medical Sciences (CIOMS).

### Generation of murine gliomas and in vivo treatments

For the RCAS-mediated gliomagenesis, newborns or 4–6-week-old mice were injected intracranially with 4×10^5^ DF1 cells 1:1 dilution between RCAS-PDGFB and RCAS-gRNA expressing cells per mouse. For the Bcan-Ntrk1-mediated gliomagenesis, *Gtv-a; hGFAP-Cre; LSL-Cas9; p53*^*lox/lox*^ newborn mice were injected intracranially with 5×10^5^ DF1 cells expressing the RCAS-Bcan-Ntrk1-gRNA-pair-2 vector. For the *p53-null* TVA-Cas9 NSCs infected with the RCAS-gRNA-pairs or the pKLV-Braf-V637E-HDR, 4–5- week-old immunodeficient *NOD/*SCID mice were injected intracranially with 5×10^5^ cells. Adults mice were anaesthetized by 4% isofluorane and then injected with a stereotactic apparatus (Stoelting) as previously described^18^. For the Cas9-inducible tumor model (*Ntv-a; LSL-Cas9*; *hUBC-CreERT2)*, 2 weeks after DF1 RCAS-gRNA plasmid injection, mice either received intraperitoneal injections of 4-Hydroxytamoxifen (Sigma-Aldrich, Cat. H6278) (2 mg/injection, 4–6 injections) or fed ad libitum with tamoxifen-containing pellets. After intracranial injection, mice were checked until they developed symptoms of disease (lethargy, poor grooming, weight loss, macrocephaly).

For in vivo studies, dabrafenib was dissolved in a mixture 0.5% hydroxypropyl methyl cellulose and 0.2% Tween 80. The drug was administered daily by oral gavage at 60 mg/kg.

### Statistical analysis

Data in bar graphs are presented as mean and SD, except otherwise indicated. Results were analyzed by unpaired two-tailed Student’s *t* tests using the R programming language. Kaplan–Meier survival curve were produced using the “survminer” R package and *P* values were generated using the Log-Rank statistic. Box-plots were made with the “ggplot2” R package. Drug dose response curves were produced with GraphPad Prism.

### Data availability

The RNA-Seq data of the various RCAS-gRNA models have been deposited in the NCBI GEO database under the accession code GSE110700. RNA-seq data for human normal brain samples were downloaded from the GTEx data portal (https://www.gtexportal.org/). ENCODE mouse brain expression data were downloaded from the NCBI (https://www.ncbi.nlm.nih.gov/gene/). RNA-seq data for *NTRK1* in the TCGA GBMLGG dataset were downloaded from the GlioVis data portal^72^ (http://gliovis.bioinfo.cnio.es). Sample IDs of patients carrying *NTRK1* gene fusions were either previously described^39^ or obtained from the TCGA Fusion gene Data Portal (http://www.tumorfusions.org). The Oncoprint for the copy number alterations of the TSG and PDGFR genes (Supplementary Figure 2a) were downloaded from the cBioportal^73^ (http://www.cbioportal.org). All the other data supporting the findings of this study are available within the article and its supplementary information files and from the corresponding author upon reasonable request.

## Electronic supplementary material

Supplementary Information

Description of Additional Supplementary Files

Supplementary Data 1

Supplementary Data 2

Supplementary Data 3
