# Supplementary material for: Somatic genome editing with the RCAS-TVA-CRISPR-Cas9 system for precision tumor modeling
Source: Nat Commun. 2018 Apr 13;9:1466. doi: 10.1038/s41467-018-03731-w (PMC5899147; doi:10.1038/s41467-018-03731-w)
Supplement: Supplementary file 1 — Supplementary Information [file 41467_2018_3731_MOESM1_ESM.pdf]

Supplementary Information

Somatic genome editing with the RCAS/TVA-CRISPR/Cas9 system for precision tumor modelling

Oldrini et al.

a

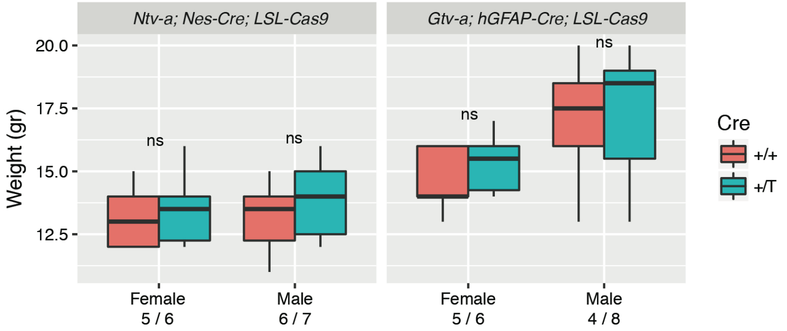

b

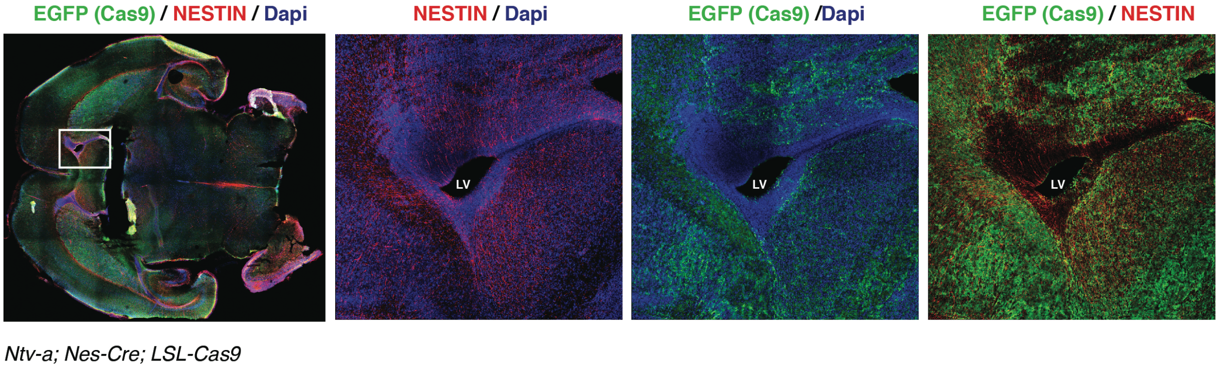

Supplementary Figure 1: CNS-specific TVA-Cas9 mice have normal development.
(a) Plots showing the weight of 4 weeks old mice of the indicated genotype and gender. CNS-specific Cas9 expressing mice presented no abnormalities in development and size. Student's *t* test: ns, not significant. The upper and lower hinges correspond to the first and third quartiles (the 25th and 75th percentiles). The upper whisker extends from the hinge to the highest value that is within 1.5 * IQR of the hinge, where IQR is the inter-quartile range. The lower whisker extends from the hinge to the lowest value within 1.5 * IQR of the hinge. **(b)** Immunofluorescence staining performed on a horizontal brain section of 2 days old *Ntv-a; Nes-Cre; LSL-Cas9* mouse with antibody against EGFP (Cas9) and NESTIN. *Left panel*: whole brain section; *right panels*: higher magnification of the left panel inset. LV, lateral ventricle.

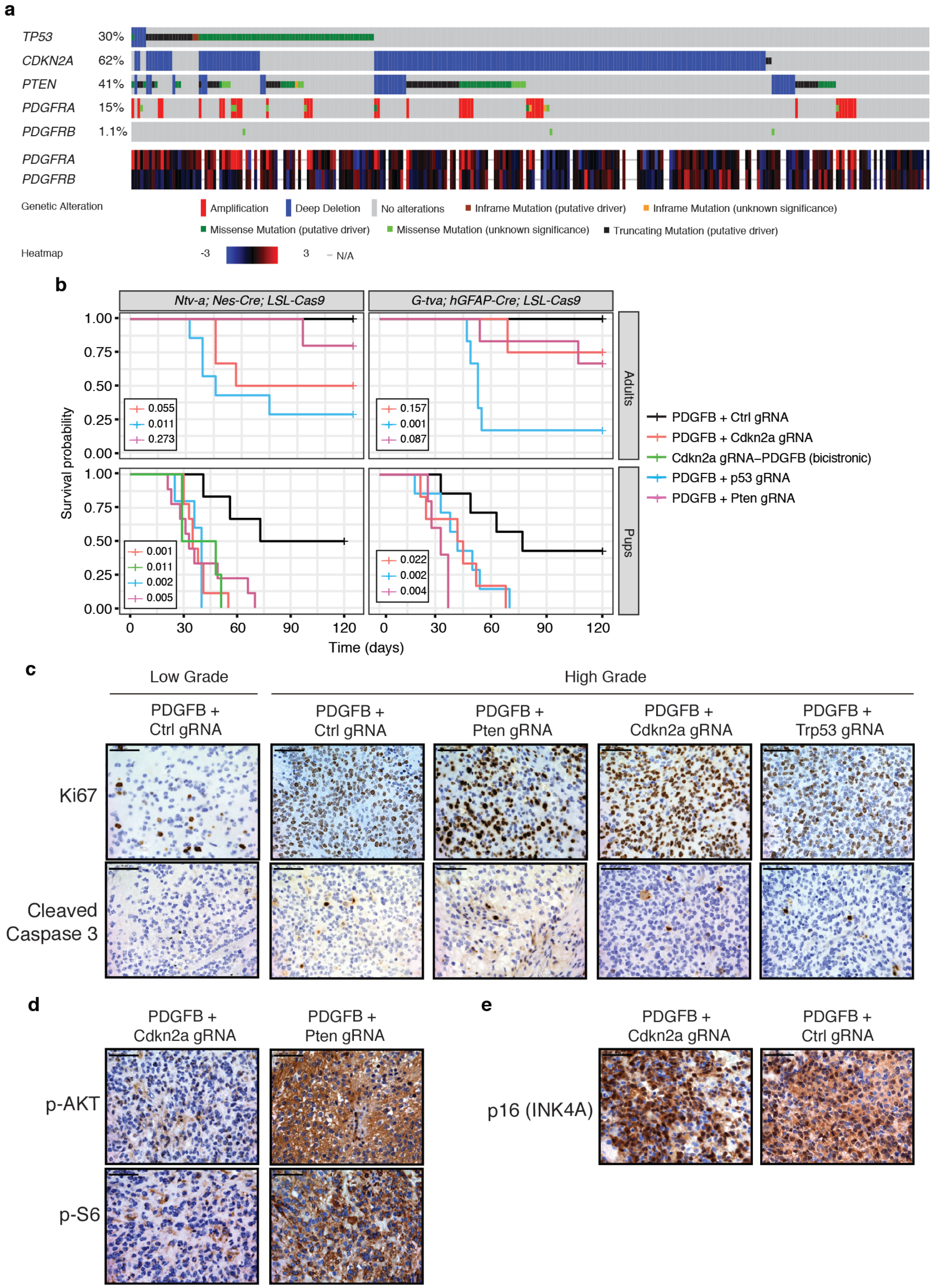
a
TP53 30%
CDKN2A 62%
PTEN 41%
PDGFRA 15%
PDGFRB 1.1%
PDGFRA
PDGFRB
Genetic Alteration
Amplification
Deep Deletion
No alterations
Inframe Mutation (putative driver)
Inframe Mutation (unknown significance)
Missense Mutation (putative driver)
Missense Mutation (unknown significance)
Truncating Mutation (putative driver)
Heatmap
-3
3
N/A
b
Ntv-a; Nes-Cre; LSL-Cas9
G-tva; hGFAP-Cre; LSL-Cas9
Adults
Pups
Survival probability
Time (days)
0.055
0.011
0.273
0.157
0.001
0.087
0.001
0.011
0.002
0.005
0.022
0.002
0.004
PDGFB + Ctrl gRNA
PDGFB + Cdkn2a gRNA
Cdkn2a gRNA–PDGFB (bicistronic)
PDGFB + p53 gRNA
PDGFB + Pten gRNA
c
Low Grade
High Grade
PDGFB + Ctrl gRNA
PDGFB + Ctrl gRNA
PDGFB + Pten gRNA
PDGFB + Cdkn2a gRNA
PDGFB + Trp53 gRNA
Ki67
Cleaved Caspase 3
d
PDGFB + Cdkn2a gRNA
PDGFB + Pten gRNA
p-AKT
p-S6
e
PDGFB + Cdkn2a gRNA
PDGFB + Ctrl gRNA
p16 (INK4A)

Supplementary Figure 2: RCAS-gRNAs against different tumor suppressor genes accelerate glioma formation.

(a) *Top panel,* Genetic alterations of *TP53*, *CDKN2A*, *PTEN*, *PDGFRA* and *PDGFRB* in GBM tissues from TCGA dataset are shown. Data (n= 273) were extracted from TCGA GBM project through the cBioPortal. *Bottom panel,* Heatmap for PDGFRA and PDGFRB mRNA expression (U133 microarray). **(b)** Kaplan-Meier survival curves of *Ntv-a; Nes-Cre; LSL-Cas9* and *Gtv-a; hGFAP-Cre; LSL-Cas9* mice injected with the indicated RCAS plasmids (see Fig. 2c for the number of mice for each group). Insets in the lower left corners show the *P* values of log-rank survival analysis, as compared to the mice injected with the RCAS-PDGFB + RCAS-Ctrl gRNA. **(c)** IHCs for Ki67 (top panels) and Cleaved caspase 3 (bottom panels) on the indicated tumors **(d)** IHCs for p-AKT (top panels) and p-S6 (bottom panels) on the indicated tumors. **(e)** IHCs for p16 (INK4A) on the indicated tumors. Scale bars: 50 μm.

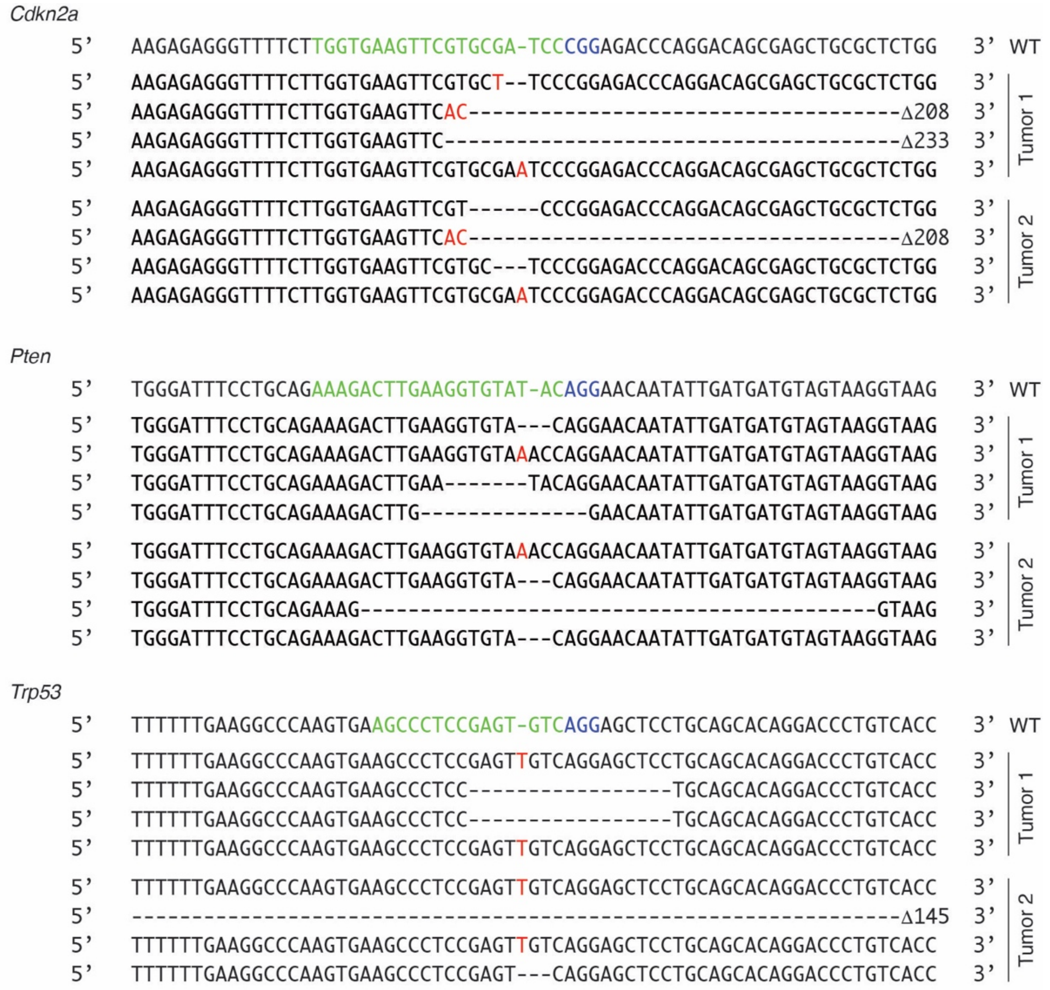

Supplementary Figure 3: CRISPR/Cas9 editing in the targeted tumor suppressor genes. The targeted loci were amplified from tumor-derived neurospheres. The PCR products were ligated into a cloning vector and sequenced via Sanger sequencing ($n = 2$ tumors, 4 clones each). Deletions or insertions were found in all sequences and are displayed as dashes and red nucleotides, respectively. The gRNA target region is displayed in green and the 3′-PAM sequence in blue.

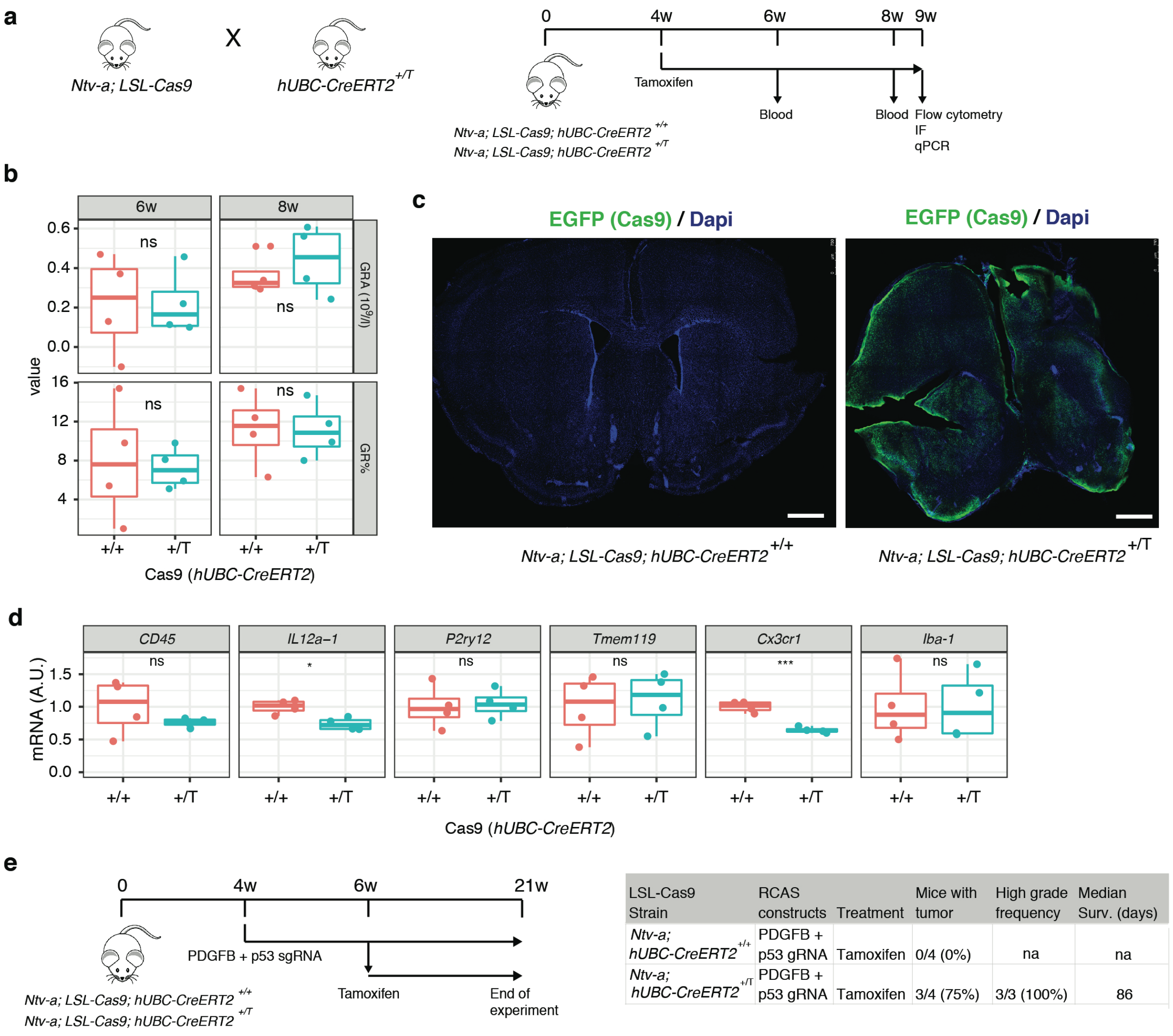

Supplementary Figure 4: Cas9 expression in adult mice doesn't induce a robust immune response.

(a) Schematic representation of the mice crossing and experimental timeline: 4 weeks old *Ntv-a; LSL-Cas9; hUBC-CreERT2* mice were treated with tamoxifen in the diet for 5 weeks. At the end of the experiment (week 9), blood, spleen and brain tissues were harvested and analyzed by flow cytometry, real-time quantitative PCR (qPCR) and immunofluorescence. **(b)** Granulocytes percentage and total number in peripheral blood of tamoxifen-treated *Ntv-a; LSL-Cas9; hUBC-CreERT2* mice of the indicated genotype. The upper and lower hinges correspond to the first and third quartiles (the 25th and 75th percentiles). The upper whisker extends from the hinge to the highest value that is within 1.5 * IQR of the hinge, where IQR is the inter-quartile range. The lower whisker extends from the hinge to the lowest value within 1.5 * IQR of the hinge. **(c)** Immunofluorescence staining performed at week 9 on brain sections of tamoxifen-treated mice with antibody against EGFP (Cas9). Scale bars: 500 µm. **(d)** qPCR analysis on a panel of microglia activation specific markers showed no major differences in mRNA expression of tamoxifen-treated *Ntv-a; LSL-Cas9; hUBC-CreERT2* mice of the indicated genotype (n = 4 per group). Student's *t* test: $***P < 0.001$; $*P < 0.05$; ns, not significant. **(e)** *Left panel:* Schematic

representation of the experimental timeline: 4 weeks old *Ntv-a; LSL-Cas9; hUBC-CreERT2* mice were injected with RCAS-PDGFB + RCAS-Trp53-gRNA. Two weeks after they were start to be treated with tamoxifen in the diet (see main text for details). *Right panel:* Table summarizing the injections performed in the *Ntv-a; LSL-Cas9; hUBC-CreERT2* mice.

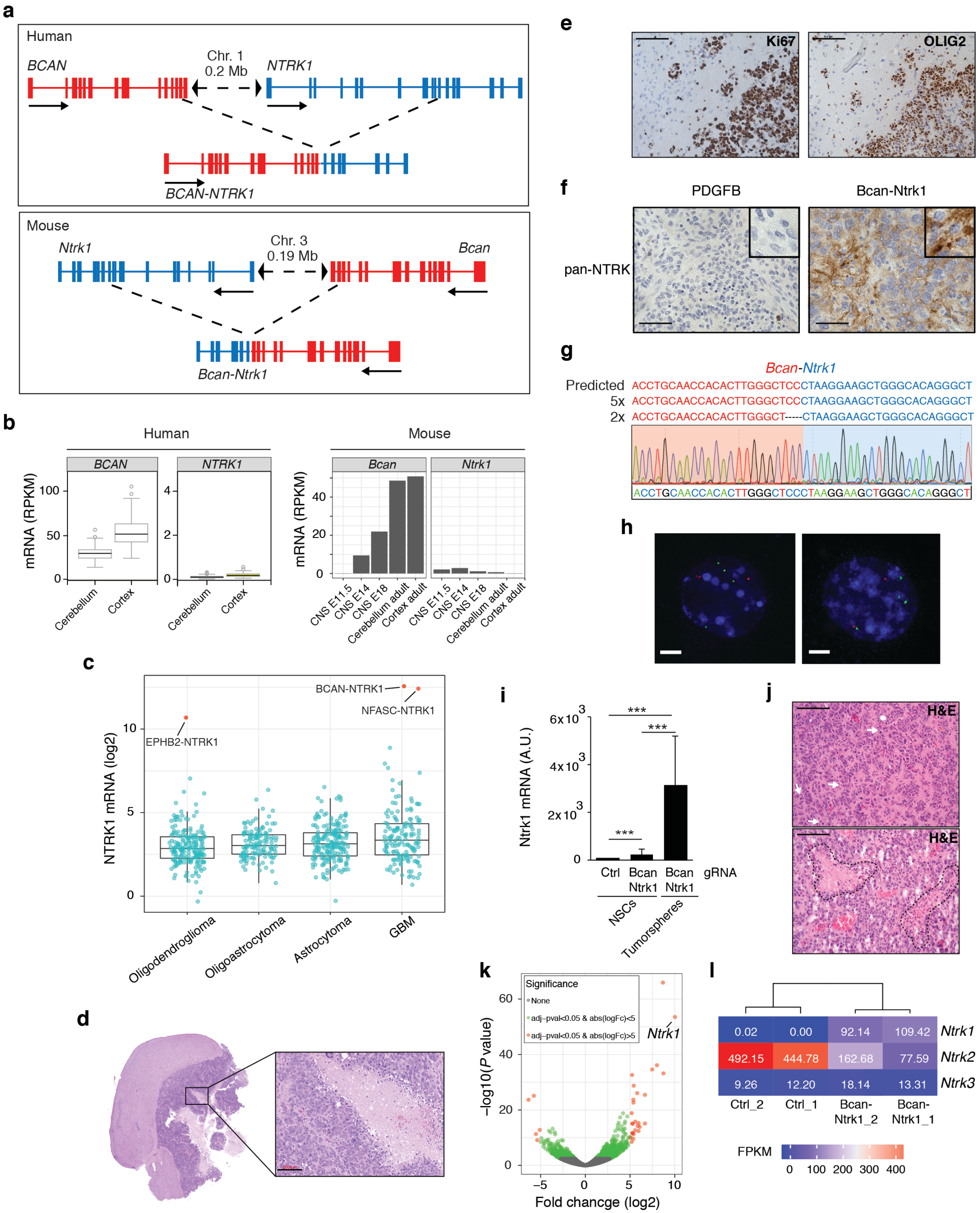

Supplementary Figure 5: Characterization of the *Bcan-Ntrk1* driven gliomas.
(a) Schematic representation of *BCAN* and *NTRK1* loci and the resulting *BCAN-NTRK1* fusion in human and mouse. **(b)** RNA-seq data for *BCAN* and *NTRK1* of human (GTEx) and mouse

(ENCODE) brain samples. RPKM, Reads Per Kilobase Million. **(c)** RNA-seq data for *NTRK1* in the TCGA glioma dataset (GBMLGG). Red dots show patients carrying the indicated *NTRK1* gene fusions. The upper and lower hinges correspond to the first and third quartiles (the 25th and 75th percentiles). The upper whisker extends from the hinge to the highest value that is within 1.5 * IQR of the hinge, where IQR is the inter-quartile range. The lower whisker extends from the hinge to the lowest value within 1.5 * IQR of the hinge. **(d)** H&E staining of a Bcan-Ntrk1 driven tumor. Inset show an example of a necrotic area. Scale bar: 100 µm. **(e)** IHCs showing clusters of OLIG2/ KI67 positive proliferative tumor cells infiltrating the normal brain parenchyma. Scale bar: 100 µm. **(f)** IHCs using pan-Trk antibody show positivity in *Bcan-Ntrk1* but not in PDGFB-driven tumors. Scale bars: 50 µm. **(g)** A PCR performed on genomic DNA extracted from a *Bcan-Ntrk1* tumorsphere line was sub-cloned and analyzed by Sanger sequencing. The sequences of 7 independent clones and a representative chromatogram are shown. **(h)** Representative FISH images using a two-color probe designed to detect the *Ntrk1-Bcan* intergenic microdeletion. The control green signal was used to count the number of chromosomes 3. The loss of two of the red signals indicates the microdeletions. To note that the presence of 4 green signals/cell suggests a possible polyploid nature of the tumor cells. Scale bar: 5 µm. **(i)** qPCR analysis, with the Ntrk1_3'-Fw and Ntrk1_3'-Rev primers, showing the upregulation of the Ntrk1 mRNA in the *Bcan-Ntrk1* tumorspheres. Student's *t* test: ***$P < 0.001$. **(j)** H&E staining of a *Bcan-Ntrk1* driven tumor obtained by *in vivo* delivery of the RCAS-Bcan-Ntrk1-gRNA plasmid into *Gtv-a; hGFAP-Cre; LSL-Cas9;* $p53^{lox/lox}$ pups. Please note the frequency of mitotic figures (*top panel,* white arrows) and the presence of necrotic areas (*bottom panel,* dashed lines), histological markers of high-grade gliomas. **(k)** Volcano plot of differential gene expression in *Bcan-Ntrk1* tumorspheres (n = 2) versus Ctrl gRNA p53 *null* cells (n = 2). **(l)** Heatmap showing FPKM values for *Ntrk1*, *Ntrk2* and *Ntrk3*. Bcan-Ntrk1_1 and Bcan-Ntrk1_2 were, respectively, derived from the NSCs transplantation and RCAS-Bcan-Ntrk1-gRNA direct injection models. FPKM, Fragments Per Kilobase of transcript per Million mapped reads.

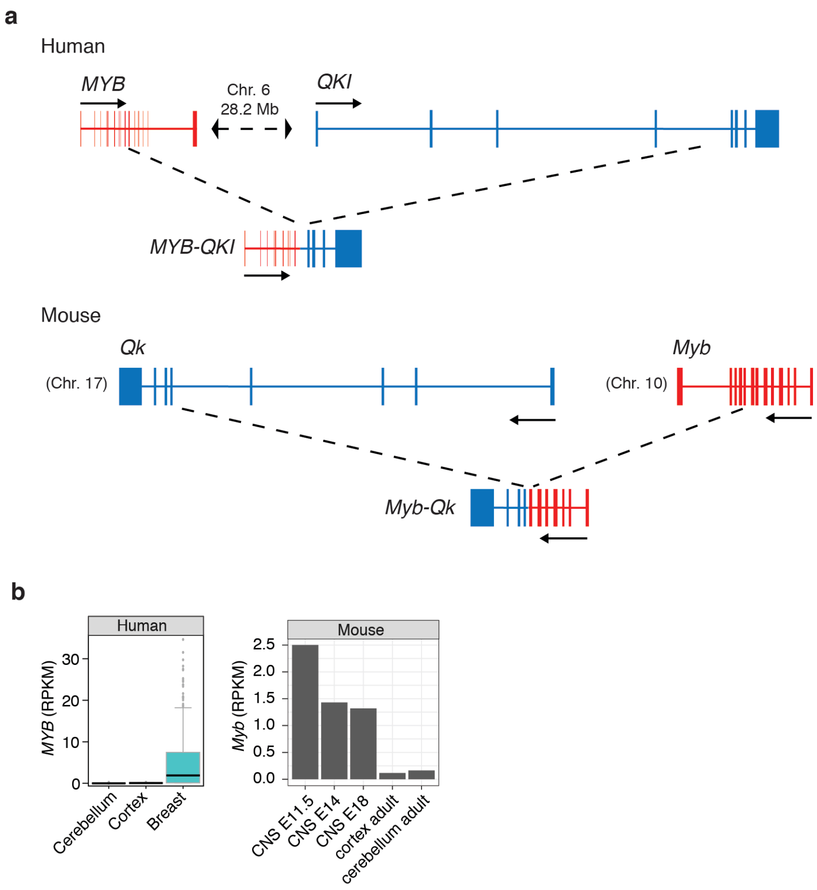

Supplementary Figure 6: MYB and QK loci and mRNA expression.
(a) Schematic representation of *MYB* and *QKI* loci and the resulting *MYB-QKI* fusion in human and mouse. **(b)** RNA-seq data for *MYB* of human (GTEx) and mouse (ENCODE) brain samples. Breast tissue samples were included as expression control for the human samples. RPKM, Reads Per Kilobase Million.

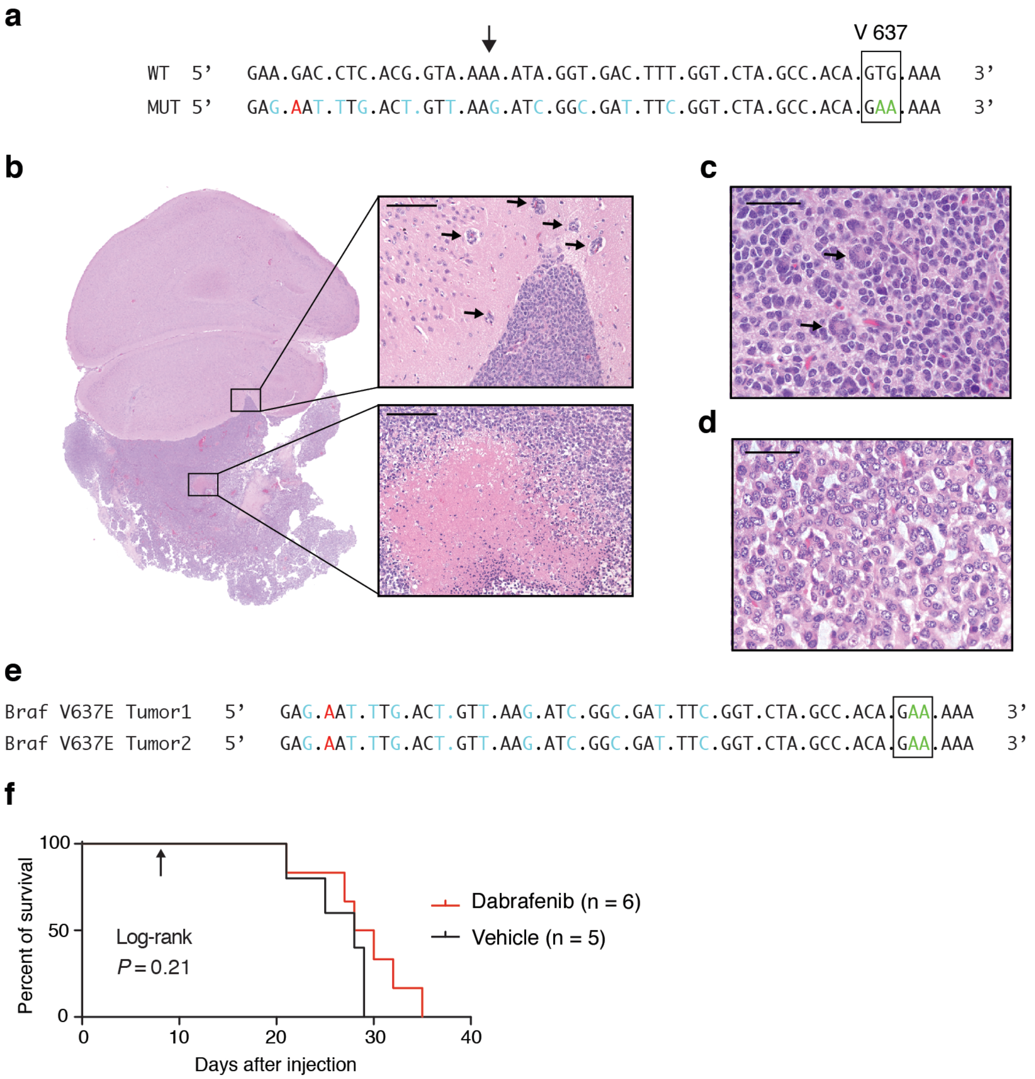

Supplementary Figure 7: Characterization of a *Braf*^{V637E} driven gliomas.
(a) Representative Illumina sequencing reads of the *Braf* locus, on genomic DNA extracted from the *p53-null* TVA-Cas9 NSCs transduced with the plasmid carrying the Braf gRNA and the *Braf*^{V637E} Homology-Directed-Repair (HDR) donor. In blue are the synonymous substitutions; in red is the conservative D624N mutation and in green is the desired V637 mutation. Black arrow indicates the site of the cut by Cas9. The black box shows the V637 residue. **(b)** H&E of a *Braf*^{V637E} induced tumors. Insets show necrotic area (*bottom panel*) and tumor infiltrating cells surrounding the vessels in the normal brain parenchyma (black arrows) (*top panel*). Scale bars: 100 µm. **(c)** H&E of a *Braf*^{V637E} induced tumors showing giant cells (black arrows). Scale bar: 50 µm. **(d)** H&E of a *Braf*^{V637E} induced tumors area with epithelioid morphology: cells with rounded cytoplasmic contours and eosinophilic cytoplasm, displaying lack of cohesion and cell-to-cell contact. Scale bar: 50 µm. **(e)** Sequencing analysis, confirming that the tumorsphere lines isolated from the *Braf*^{V637E} induced tumors carried the V637E mutation (black box) in 100% (10/10) analyzed clones. **(f)** Kaplan Meier survival curves of *NOD/SCID* mice intracranially injected with *Braf*^{V637E} tumorspheres. Seven days after injection, the mice were randomly separated in two groups and treated daily with either vehicle or dabrafenib (60 mg/kg).

Fig. 2b

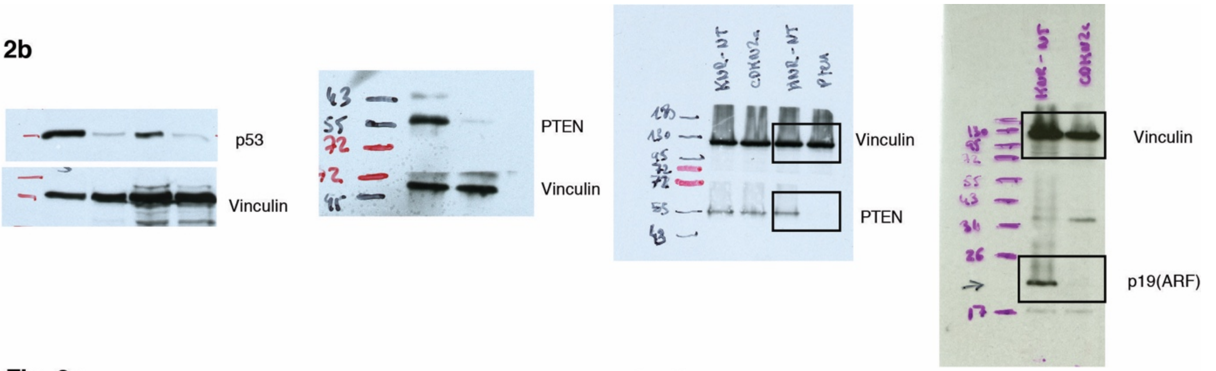

Fig. 2e

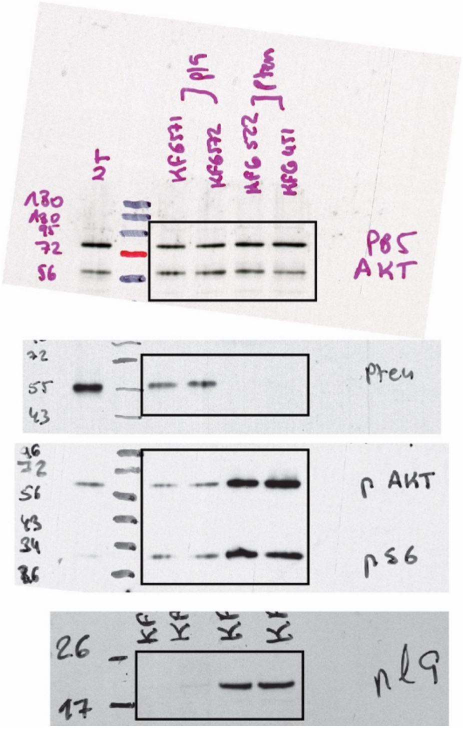

Fig. 2g

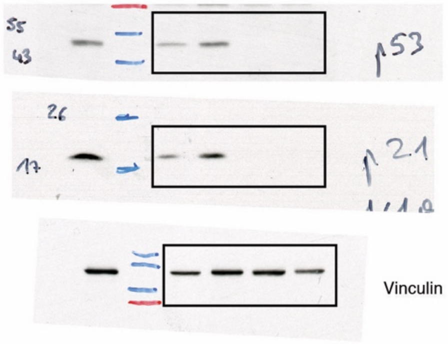

Supplementary Figure 8: Uncropped western blot images for Figure 2

Fig. 5c

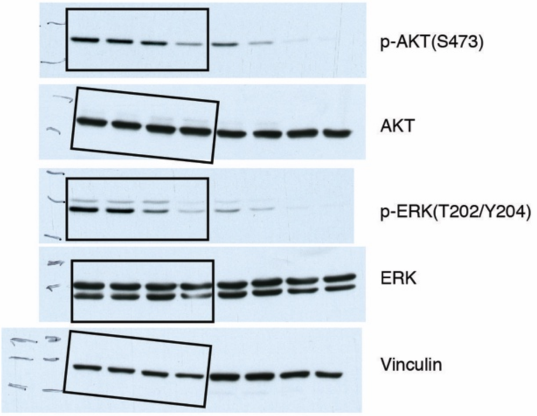

Fig. 5g

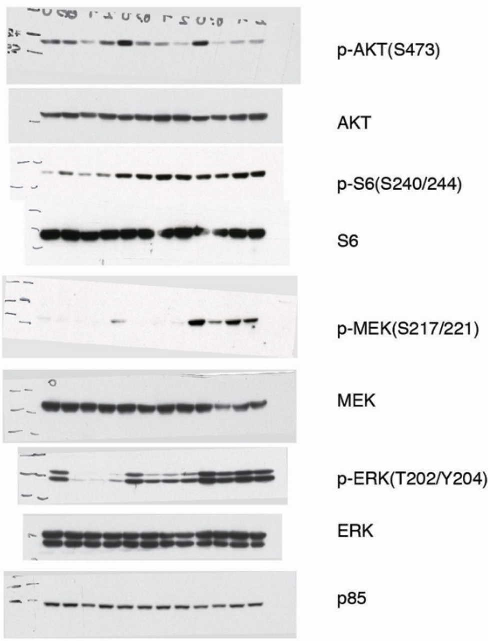

Fig. 7e

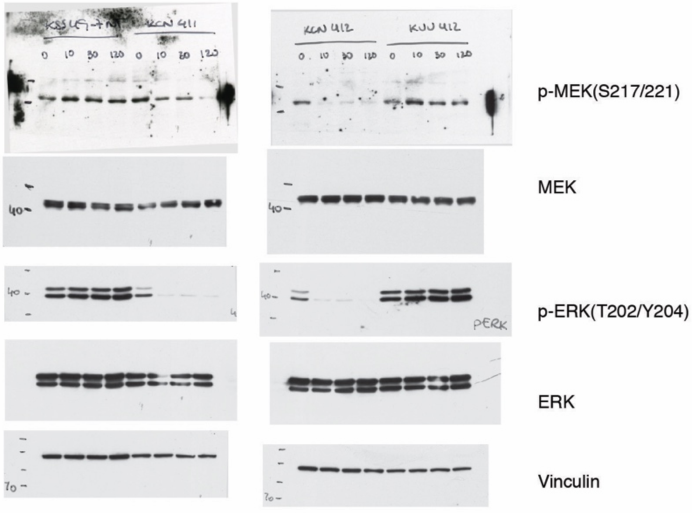

Fig. 7g

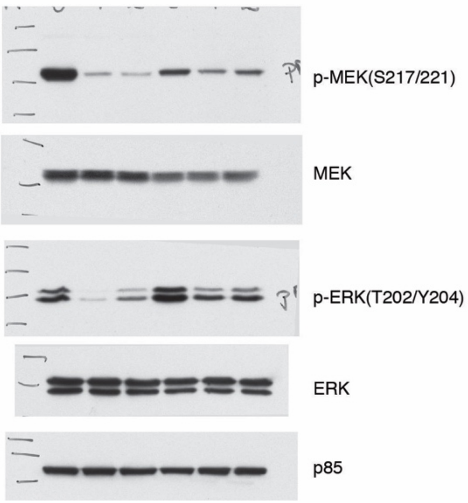

Supplementary Figure 9: Uncropped western blot images for Figures 5 and 7

Supplementary Table 1. ssGSEA GBM subtype classification

Sample	ssGSEA.subtype.call	Proneural	Classical	Mesenchymal	Proneural_pval	Classical_pval	Mesenchymal_pval
PDGFB_Cdkn2a_ko_1	Classical	3075.36	6770.61	1116.59	0.881	0.0919	0.952
PDGFB_Cdkn2a_ko_2	Classical	3652.36	6692.01	409.33	0.254	0.15	1
PDGFB_Pten_ko_1	Classical	2770.98	7040.85	1282.43	0.99	0.00899	0.825
PDGFB_Pten_ko_2	Classical	2554.11	6831.98	906.66	0.998	0.0579	0.989
PDGFB_Trp53_ko_1	Proneural	4277.75	5443	1490.44	0.00599	1	0.528
PDGFB_Trp53_ko_2	Proneural	4564.98	5810.29	959.25	0.000999	0.997	0.987

Note: Subtype calls with Enrichment scores (ES) and empirical p-values (pval) are shown

Supplementary Table 2. Tophat-fusion data for the Bcan-Ntrk1 and Myb-Qk fusions

Sample_ID	Gene_left	Chr_left	Coordinate_left	Gene_right	Chr_right	Coordinate_right	Spanning_reads	Spanning_mate_pairs
Bcan_Ntrk_1	Ntrk1	chr3	87586848	Bcan	chr3	87792006	125	23
Bcan_Ntrk_2	Ntrk1	chr3	87586848	Bcan	chr3	87791994	2	2
Myb_Qk_1	Myb	chr10	20865991	Qk	chr17	10410667	5	2
Myb_Qk_2	Myb	chr10	20865991	Qk	chr17	10410667	34	8
Myb_Qk_2	Qk	chr17	10431757	Myb	chr10	20862897	19	14

Legend

1. Sample_ID: Sample name in which a fusion is identified
2. Gene_left: Gene on the "left" side of the fusion
3. Chr_left: Chromosome ID on the left
4. Coordinate_left: Coordinates on the left
5. Gene_right: Gene on the "right" side of the fusion
6. Chr_right: Chromosome ID on the right
7. Coordinate_right: Coordinates on the right
8. Spanning_reads: Number of spanning reads - this is the number of reads that span a fusion point all on their own. In other words, the read itself has a fusion break point within it.
9. Spanning_mate_pairs: Number of spanning mate pairs - this is the number of pairs of reads where one read maps entirely on the left and the other read maps entirely on the right of the fusion break point. Neither read is split, so these pairs are not counted at all in (8).

Supplementary Table 3. Primers list

**Primer name**	**Sequence 5' → 3'**
aTTB-Fw	GGGGACAAGTTTGTACAAAAAAGCAGGCTTAATC CAGTTTGGTTAGTACCGGG
aTTB-Rv	GGGGACCACTTTGTACAAGAAAGCTGGGTTTCTTT TAAAAAGTGGCTAAGATCTACAGCTGC
pDONR_BbsI_mut-Fw	AAAACGAAAGGCCCAGTTTTCCGACTGAGCCTTTC
pDONR_BbsI_mut-Rv	GAAAGGCTCAGTCGGAAAACTGGGCCTTTCGTTTT
pDONR-Fw	GCGGCCGCTAGGTACCT
pDONR-Rv	TGGGCCAGGATTCTCCTCCA
PDGFB-Fw	TCTCCTAACATGCGGTGACGTGGAGGAGAATCCT GGCCCAATGAATCGCTGCTGGGC
PDGFB-Rv	GCCTTGTAAGTCATTGGTCTTAAAGGTACCTAGCG GCCGCCTAGGCATAATCTGGCACGTCATA
PDGFB_BbsI_mut-Fw	AGGCCACGGTGACGCTGGAGGATCACCTGGCATG CAAGTG
PDGFB_BbsI_mut-Rv	CACTTGCATGCCAGGTGATCCTCCAGCGTCACCGT GGCCT
Trp53-sgRNA-Fw	CACCCGAGTGAAGCCCTCCGAGTGTCGT
Trp53-sgRNA-Rv	TAAAACGACACTCGGAGGGCTTCACTC
Braf-sgRNA-Fw	CACCGAGTCACCTATTTTTACCGTGGT
Braf-sgRNA-Rv	TAAAACCACGGTAAAAATAGGTGACT
Cdkn2a-sgRNA-Fw	CACCGTGGTGAAGTTCGTGCGATCCGT
Cdkn2a-sgRNA-Rv	TAAAACGGATCGCACGAACTTCACCA
Pten-sgRNA-Fw	CACCGAAAGACTTGAAGGTGTATACGT
Pten-sgRNA-Rv	TAAAACGTATACACCTTCAAGTCGGGC
Ctrl-sgRNA-Fw	CACCGTGCTTTTCCGACACGGTCGCGT
Ctrl-sgRNA-Rv	TAAAACGCGACCGTGTCGGAAAAGCA
Trp53_seq-Fw	GGAGGCCAGCCTGGGATAAG
Trp53_seq-Rv	CACGAAAGACAACTCCCCGG
Cdkn2a_seq-Fw	ATGGGCGTGGAGCAAAGATG
Cdkn2a_seq-Rv	TAAGCCGAAGGGGGAAAGCG
Pten_seq-Fw	GCCTCAGTCGCGTATTCTG
Pten_seq-Rv	CATCCAGTGACGCATCCAG
Bcan-Ntrk1-gRNA-pair-1	ATGCGAGAAAAGCCTTGTTTGgCCGGCTAGCTGAG ATTGCTAGTTTTAGGGTCTTCGAGAAGACCTCACC gAACCACACTTGGGCTCCTAAGTTTTAGAGCTAGA AATAG
Bcan-Ntrk1-gRNA-pair-2	ATGCGAGAAAAGCCTTGTTTGgCCGGCTAGCTGAG ATTGCTAGTTTTAGGGTCTTCGAGAAGACCTCACC gAACCACACTTGGGCTCCTAAGTTTTAGAGCTAGA AATAG
Myb-Qk-gRNA-pair	ATGCGAGAAAAGCCTTGTTTGgTTCCCTCAATCTA ACTACAAGTTTTAGGGTCTTCGAGAAGACCTCACC

	GAACTTCATTAATTCACACCGTTTTAGAGCTAGAAATAG
F5	TTTGAGACTATAAATATGCATGCGAGAAAAGCCTTGTTTG
R1	GACTAGCCTTATTTTAACTTGCTATTTCTAGCTCTAAAAC
Bcan-A	CTATGGAAGACAAACACACCCATCCTC
Bcan-B	GTAAGTGACAGGAGGAAACAGGTAGG
Ntrk1-C	CAGAGACCTAGAAGGAGGGTCAGG
Ntrk1-D	GCACATCAAGAGACCCAGTGGA
Bcan-Ntrk1 cDNA Fw (Bcan-Fw)	GGAAGAGGGCGGCAGAGAC
Bcan-Ntrk1 cDNA Rv (Ntrk1-Rev)	GGTCGCCCTCGGCTGC
Ntrk1_3'-Fw	TATGGAAAGCAGCCCTGGTA
Ntrk1_3'-Rv	CTTCATGCTGAGGCGTTG
Qk-A	GTGCATATACCATGTGACTTTGAACTACAAAAGT
Myb-B	GTAGAATTGCTAAGGCACGTTGATGTTTC
Myb-Qk cDNA Fw (Myb-Fw)	GTGCCTCACCAGCAAGGTG
Myb-Qk cDNA Rv (Qk-Rev)	GTGATTTAATGTTGGCGTCTCTGTAGG
Myb_5'-Fw	GACCCCGACACAGCATCTAC
Myb_5'-Rv	GTTCCACCAGCTTCTTCAGC
Braf_cds_Fw	CAGACTGCACAGGGCATGG
Braf_cds_Rv	GTTTGAATAAGGTAGCTGGCCGG
GAPDH-Fw	CGTCCCGTAGACAAAATGGT
GAPDH-Rv	TCAATGAAGGGGTCGTTGAT
CD45-Fw	TCATGGTCACACGATGTGAAGA
CD45-Rv	AGCCCGAGTGCCTTCCT
IL12a-1-Fw	CCATCAGCAGATCATTCTAGACAA
IL12a-1-Rv	CGCCATTATGATTCAGAGACTG
P2ry12-Fw	CAAGGGGTGGCATCTACCTG
P2ry12-Rv	AGCCTTGAGTGTTTCTGTAGGG
Tmem119-Fw	GTGTCTAACAGGCCCCAGAA
Tmem119-Rv	AGCCACGTGGTATCAAGGAG
Cx3cr1-Fw	CAAATTCTCTAGATCCAGTTCAGG
Cx3cr1-Rv	AAGTTCCCTTCCCATCTGCT
Iba-1-Fw	GGATTTGCAGGGAGGAAAAG
Iba-1-Rv	TTCCATTCAGATCAAACTCC
Erbb2-Fw	CTTTGATGGGAACCCCTCCT
Erbb2-Rv	ATCATGGAGAATCCGTCCCC
Cdk6-Fw	CATCGTGATCTGAAACCGCA
Cdk6-Rv	CGAGGTAAGGGCCATCTGAA
p21-Fw	TCCACAGCGATATCCAGACA
p21-Rv	GGACATCACCAGGATTGGAC
Puma-Fw	ACGACCTCAACGCGCAGTA
Puma-Rv	CGGTGTCGATGCTGCTCTT
Mdm2-Fw	GGAAGATGCGCGGGAAGTA

Mdm2-Rv	CCGCTCGCCCAGCAG
Noxa-Fw	AGGAAGGAAGTTCCGCCG
Noxa-Rv	AGCGTTTCTCTCATCACATCACA
P7Fw-Idx20	CAAGCAGAAGACGGCATACGAGATggccacGTGACTGGAGTTCAGACGTGTGCTCTTCCGATCtactgcacctcagatGtatttcttcatg
P5Rev	AATGATACGGCGACCACCGAGATCTgactaaaggtgtgcactactaagc
Braf_seq	CTGATGGGACCCACTCCACCGAGA
