# Supplementary material for: Somatic genome editing with the RCAS-TVA-CRISPR-Cas9 system for precision tumor modeling
Source: Nat Commun. 2018 Apr 13;9:1466. doi: 10.1038/s41467-018-03731-w (PMC5899147; doi:10.1038/s41467-018-03731-w)
Supplement: Supplementary file 2 — Description of Additional Supplementary Files [file 41467_2018_3731_MOESM2_ESM.pdf]

Description of Additional Supplementary Files

File Name: Supplementary Data 1
Description: Differentially gene expression analysis for the Bcan-Ntrk1 tumors

File Name: Supplementary Data 2
Description: Differentially gene expression analysis for the Myb-Qk cells

File Name: Supplementary Data 3
Description: V637E HDR donor sequence
