# Supplementary material for: Somatic genome editing with the RCAS-TVA-CRISPR-Cas9 system for precision tumor modeling
Source: Nat Commun. 2018 Apr 13;9:1466. doi: 10.1038/s41467-018-03731-w (PMC5899147; doi:10.1038/s41467-018-03731-w)
Supplement: Supplementary file 5 — Supplementary Data 3 [file 41467_2018_3731_MOESM5_ESM.docx]

**Supplementary Data 3. V637E HDR donor sequence**

TTAATTAAGCCTGGGCCGCTTTCCCAGCAGAGTTAAAGTGTATCTGGGCCAAATCAAATTAGAACGTCCCCAGGTCCCCCATAGGCTTGGAAGTATCTTAACCTTATGCTCACATACTTCAGGCCTGGGACAGCTTAAAAGTAGGTCGTGTAAGCTAAATAGAACAAAGGTCTTGCTCAAAACATATTTGTTGACTTTCAGAGGACATACGAATCTCTGTATTTTAAAAAGAAAATATAGTACTATAGAGTTATGGAAAGTAATTCTTTTACCTGAAATCTTCAAAATGCTTTCTCTAATAGGAAAATGCAATCTGTTTTTTTTTTTTTCCTTTACTTACTGCACCTCAGATATATTTCTTCATGAGGATTTGACTGTTAAGATCGGCGATTTCGGTCTAGCCACAGaaAAATCTCGGTGGAGTGGGTCCCATCAGTTTGAACAGTTGTCTGGATCTATTTTGTGGATGGTAAGAATTGAGGAGGTATCTCAGTTGGTCACATTCTTCAGTCCTAAACTAGTGACTGCTTACGAGAAATACACTGAAGGGCTTAACTACAGTACTTTCACAGTTCCCACTACTCACAGGAATAACATTGTAAGCCAGGCATAATTGCACATGCCTTTAATCCCAGCACTTAGAAAGCTGAACAACTGGATAGACCTCTATTAGTTTGAAGCCAGCCTGTCTATATAGTTCCAGAACAGCCAGAGCTACAAAGTGAGACTTTGTCTCTTATTTTTTGTGTAAATGGTCTTTTTTCTTCTCTTTAAAAAAACTTATTGCAAAACCAGTTTCAGTATATTTTTAATTAA

V637E

Synonymous mutations

PacI restriction sites
